# Nanomaterial-Enabled Spectroscopic Sensing: Building a New Paradigm for Precision Detection of Pesticide Residues

**DOI:** 10.3390/nano15211634

**Published:** 2025-10-27

**Authors:** Mei Wang, Yue Niu, Hao Peng, Pengcheng Zhang, Quan Bu, Xianghai Song, Shouqi Yuan

**Affiliations:** 1 School of Agricultural Engineering, Jiangsu University, Zhenjiang 212013, China; 2 Institute of the Green Chemistry and Chemical Technology, School of Chemistry and Chemical Engineering, Jiangsu University, Zhenjiang 212013, China; 3 Research Center of Fluid Machinery Engineering and Technology, Jiangsu University, Zhenjiang 212013, China

**Keywords:** spectroscopic techniques, nanomaterials, pesticide residue detection, SERS, hyperspectral imaging

## Abstract

This review summarizes the application of spectroscopic techniques in pesticide residue analysis, with a focus on the principles, advancements, and challenges of surface-enhanced Raman spectroscopy (SERS), infrared spectroscopy, fluorescence spectroscopy, ultraviolet-visible (UV-Vis) spectroscopy, and hyperspectral imaging (HSI). Nanomaterials, serving as key enhancing substrates, significantly improve the sensitivity and selectivity of these detection methods. This article critically evaluates the strengths and limitations of each technique in practical applications—such as the exceptional sensitivity of SERS versus its dependence on substrate reproducibility, and the non-destructive nature of hyperspectral imaging against the complexity of data processing. Future research directions should emphasize the development of intelligent nanosubstrates, the construction of cross-modal spectral databases, and the miniaturization of integrated spectroscopic-mass spectrometric instruments. These advancements are essential for enhancing the efficiency and reliability of agricultural and food safety monitoring.

## 1. Introduction

Pesticides, as a critical technological tool for ensuring food security, have long been the subject of debate in agricultural science because of their dual nature—delivering substantial benefits while posing potential risks [1]. In practical agricultural production, pesticides play a vital role in controlling pests and diseases and suppressing weeds, thereby substantially increasing crop yields [2,3,4]. According to the Food and Agriculture Organization of the United Nations, discontinuing pesticide use globally could result in annual crop losses exceeding 30%, with some cash crops facing total failure. Beyond protecting yields, pesticides also reduce post-harvest losses and improve the visual quality of produce, thereby supporting the large-scale, mechanized development of modern agriculture [5,6]. For example, these have dramatically simplified field management, significantly boosting agricultural efficiency. However, the issue of pesticide residues has become increasingly prominent. Residual chemicals in food can accumulate through the food chain, posing potential threats to both ecosystems and human health—especially in fresh produce, where minimal processing increases exposure risks [7,8]. To address this challenge, international regulatory frameworks—centered on Maximum Residue Limits (MRLs)—have been established and are implemented through comprehensive measures, including controlled pesticide development, mandated pre-harvest intervals, and rigorous monitoring throughout the supply chain [9]. In China, innovative initiatives—such as technology outreach programs for agribusinesses and farmer training in proper pesticide use—aim to reduce over-application at the source, while green solutions, such as biodegradation technologies, are being actively explored. Against this backdrop, achieving a scientifically sound balance between agricultural productivity and ecological safety has emerged as a key challenge in advancing sustainable agriculture [10].

Pesticide residue detection serves as a critical line of defense in food safety, with the sophistication of detection methods directly influencing regulatory effectiveness [11,12,13]. Physicochemical properties—such as chemical structures and molecular weights—play crucial roles in pesticide detection, as virtually all analytical methods rely on these intrinsic characteristics. For example, mass spectrometry (MS) enables detection based on molecular weight, whereas vibrational spectroscopic techniques such as Raman and infrared spectroscopy identify pesticides through their unique molecular vibrations. These fundamental properties are readily accessed in authoritative databases such as PubChem and the Pesticide Properties Database, which provide comprehensive information to support method development and data interpretation in pesticide analysis. Conventional techniques such as chromatography and MS, while highly accurate, face practical limitations, including high equipment costs, complex sample preparation, and lengthy analysis times [14,15,16]. In contrast, spectroscopic technologies are reshaping the landscape of residue detection by enabling non-destructive, rapid, and in situ analysis capabilities [17,18]. For example, Raman spectroscopy enables on-site screening within two minutes, shifting regulatory strategies from reactive traceability to proactive interception. Aqueous-phase detection systems eliminate the need for toxic solvents, aligning with the principles of green chemistry. Notably, gold nanoparticle-enhanced Raman spectroscopy (SERS) achieves detection limits as low as 0.1 ppb, and when combined with hyperspectral imaging (HSI) and artificial intelligence (AI) algorithms, it can precisely map the spatial distribution of residues. These technological advances are transforming spectroscopic methods from supplementary tools into core analytical solutions in the industry.

Owing to their unique analytical principles, spectroscopic techniques offer clear advantages across multiple dimensions. They not only overcome the limitations of traditional methods but also continuously expand application frontiers through innovation, driving a transformative shift in modern analytical science [19]. In terms of analytical performance, spectroscopy offers three key advantages: non-contact measurement, ultra-high sensitivity, and multiplex detection capability. Unlike conventional methods, which require extensive sample preparation, spectroscopic analysis enables real-time, dynamic monitoring. Its distinctive “molecular fingerprint” identification—such as characteristic Raman peaks—is particularly effective for detecting trace analytes and analyzing complex matrices [20]. With advances in miniaturization, spectroscopic instruments are becoming increasingly portable and intelligent, opening new possibilities for rapid on-site testing [21,22].

These advantages are evident in four key areas:

(1) Non-destructive analysis: Techniques such as near-infrared (NIR) and Raman spectroscopy analyze the absorption or scattering of light at specific wavelengths, enabling in situ, non-invasive testing [23]. In agricultural applications, for example, researchers can directly scan the surfaces of fruits and vegetables using Raman spectroscopy, completing residue analysis in seconds—without the need for sample grinding or solvent extraction, as required by traditional methods. This non-destructive nature is especially valuable for delicate produce such as strawberries and grapes, preserving sample integrity while maintaining high testing efficiency and minimizing waste [24].
(2) High detection efficiency: Spectroscopic methods offer remarkable speed. While traditional chromatographic analysis may take 30 min to several hours per sample, fluorescence and surface-enhanced Raman spectroscopy (SERS) can deliver results within seconds. When integrated with automated systems, spectroscopic platforms can process hundreds of samples per hour [25]. For instance, portable NIR spectrometers, are now widely used in field monitoring, enabling farmers to accurately assess pesticide degradation and avoid residue violations due to insufficient pre-harvest intervals [26]. This high-throughput capability significantly enhances the coverage and timeliness of food safety oversight.
(3) Simultaneous multi-residue detection: Spectroscopy excels at detecting multiple pesticide residues simultaneously—a crucial advantage given the widespread use of pesticide mixtures. Traditional single-analyte methods are increasingly inadequate. In contrast, full-spectrum scanning combined with chemometric modeling enables spectroscopic techniques to identify multiple residues simultaneously. For example, Fourier-transform infrared (FTIR) spectroscopy, when coupled with principal component analysis (PCA), can clearly distinguish characteristic peaks of different pesticide classes—such as organophosphates and pyrethroids—providing an efficient solution for complex residue analysis [27]. This capability is particularly valuable in responding to sudden contamination incidents.
(4) Smart integration and cost effectiveness: Modern spectroscopic technologies have achieved significant advances in integration and affordability [28]. By incorporating miniaturized optical components and AI algorithms, next-generation devices have significantly lowered operational barriers [29]. For example, smartphone-coupled, cloud-based Raman spectrometers enable field personnel to perform tests with minimal training. Moreover, spectroscopy eliminates the need for consumables such as chromatographic columns and high-purity solvents, reducing long-term operating costs to just 20–33% of those associated with traditional methods. This economic advantage makes spectroscopic technologies particularly suitable for resource-limited rural areas and small-to-medium enterprises.

Given the growing importance and promising potential of spectroscopic analysis in pesticide residue detection, this review provides a systematic overview of various spectroscopic techniques—their principles, methodologies, strengths, and limitations. It focuses on five key technologies: SERS, infrared spectroscopy, fluorescence spectroscopy, ultraviolet-visible (UV-Vis) spectroscopy, and HSI. This review examines their fundamental principles, application characteristics, suitable scenarios, and current technical limitations while also outlining future development trends. The aim is to provide technical guidance and theoretical insights to support the optimization and broader adoption of spectroscopic detection in food safety and agricultural monitoring.

## 2. Principles and Applications of Spectroscopic Techniques

### 2.1. SERS Techniques

Raman spectroscopy is a powerful tool for probing molecular structures, based on the detection of inelastic scattering that occurs when photons interact with molecules [30]. In 1928, C.V. Raman discovered that when monochromatic light (typically from a laser) interacts a sample, in addition to elastic scattering (Rayleigh scattering), approximately 0.1% of the photons undergo a frequency shift—known as the Raman shift—producing two sets of characteristic spectral lines: Stokes lines (at lower frequency) and anti-Stokes lines (at higher frequency). This “molecular fingerprint” arises from vibrational energy level differences in chemical bonds, conferring a unique advantage to Raman spectroscopy in substance identification. For example, Dias et al. successfully constructed a comprehensive spectral database containing characteristic Raman profiles of 78 different pesticides in both liquid and solid forms. Their work provides a valuable technical reference for rapid pesticide detection. The database systematically annotates the characteristic Raman bands of each pesticide and organizes them by mode of action, chemical structure, active ingredient, and relative peak intensity, providing standardized data support for subsequent pesticide identification research. This achievement not only fills a critical gap in the application of Raman spectroscopy to pesticide analysis but also fosters collaborative innovation within the scientific community through its open-access, shared database model [31].

However, conventional Raman spectroscopy suffers from inherently weak signal intensity—typically only about 10^−6^ relative to the Rayleigh scattering signal. To overcome this limitation, significant technological advancements have been made to enhance detection capability. For instance, Fourier-transform Raman (FT-Raman) spectroscopy employs a 1064 nm NIR laser to effectively suppress fluorescence interference from samples. However, FT-Raman is susceptible to sample movement and prone to thermal drift, both of which can compromise measurement stability [32]. In contrast, surface-enhanced Raman spectroscopy (SERS) has emerged as a major research focus by leveraging localized electromagnetic field effects generated by nanostructured substrates, thereby boosting detection sensitivity to the single-molecule level [33].

#### 2.1.1. Principles

SERS has overcome the sensitivity limitations of traditional Raman spectroscopy, laying the foundation for trace detection [34,35]. SERS achieves a significant enhancement in Raman signals through interactions between metal nanostructures and target molecules, involving both physical and chemical enhancement mechanisms [36,37]. The physical enhancement results from localized surface plasmon resonance (LSPR) on the surfaces of metal nanoparticles, such as silver and gold. Upon illumination with laser light, electromagnetic “hot spots” are formed on these surfaces, boosting the Raman scattering intensity of adsorbed molecules by factors of 10^6^ to 10^14^. Chemical enhancement relies on charge transfer between the metal substrate and the molecules, forming metal-molecule complexes that alter molecular polarizability, thereby enhancing the Raman signal by approximately 10^2^ times [38,39,40]. For example, Guo et al. demonstrated an enhancement factor of 1.39 × 10^9^ using silver/graphene composite substrates optimized for nanoparticle size (around 60 nm) and distribution density, with acetone solvent promoting charge transfer between pesticide molecules and the substrate. Typical chemical enhancement factors range from 10^1^ to 10^3^, but they are sensitive to molecular orientation. When both mechanisms work synergistically (e.g., molecules adsorbed in hotspots of Au@Ag core–shell structures), the enhancement factor can reach 10^10^ to 10^14^, enabling single-molecule detection. Transition metal oxides (such as TiO_2_) can also produce significant chemical enhancement through interface charge transfer involving defect energy levels [41].

In the detection of pesticide molecules, SERS demonstrates unique advantages [42,43]. By leveraging specific adsorption interactions between metal nanosubstrates and pesticide molecules, along with probe molecules (such as paraquat) to create a “bridging” model, SERS can capture weakly Raman-active organochlorine pesticides and excite identifiable signals [44]. In practical applications, flower-like silver nanoparticles (AgNPs) substrates maintain pesticide structural integrity within a pH range of 6.66 to 11.11. Combined with droplet concentration methods, detection limits can be reduced to sub-ppb levels (e.g., 0.1 ppb), meeting the requirements for detecting trace residues in complex matrices like tea [15,45]. Compared to chromatographic methods, SERS does not require complex sample preparation, with each analysis test taking just a few seconds. Additionally, it is capable of distinguishing isomers through characteristic peak shifts. As a non-destructive detection technique, SERS not only meets increasingly stringent food safety standards (such as the EU limit of 0.01 mg/kg) but also provides an efficient and portable tool for monitoring the quality and safety of agricultural products.

#### 2.1.2. SERS Substrates and Signal Enhancement Strategies

The performance of SERS technology depends critically on the rational design and optimization of substrate materials. Based on different enhancement mechanisms and material properties, contemporary mainstream SERS substrates can be categorized into three primary types:

Precious metal substrates, including gold and silver nanostructures (such as nanospheres, nanorods, nanocubes, etc.), which offer enhancement factors ranging from 10^6^ to 10^8^. However, these materials are prone to oxidation and are relatively expensive [46,47].

Semiconductor substrates (such as TiO_2_, MoO_3−x_), which achieve charge transfer enhancement through defect engineering to control oxygen vacancies. Although their enhancement factors are lower (10^2^ to 10^4^), they exhibit excellent chemical stability [48,49].

Composite functional substrates, including core–shell structures (such as Au@SiO_2_), flexible substrates (such as PE film loaded with nanoparticles), and magnetic composite microspheres (such as Fe_3_O_4_@Au) [50,51].

Research indicates that the performance of SERS substrates is primarily influenced by three key factors: surface morphology, size parameters, and structural design. In terms of morphology, spherical nanoparticles exhibit uniform LSPR intensity, while polyhedral structures such as rods and cubes show angle-dependent enhancement effects [52]. Size effects demonstrate that when the size of AgNPs increases from 50 nm to 100 nm, electromagnetic field coupling significantly enhances, leading to increased SERS signal intensity. Regarding structural design, three-dimensional array substrates fabricated using nanoimprint lithography can achieve enhancement factors in the range of 10^7^ to 10^8^. The unique short-range island distribution feature of these substrates effectively increases hotspot density and signal uniformity [53].

In summary, optimizing the performance of SERS substrates requires a comprehensive consideration of three critical factors: morphology (ranging from uniform spheres to anisotropic polyhedra), size (with optimal electromagnetic coupling typically observed between 50–100 nm), and structural design (particularly high hotspot density in three-dimensional arrays). These research findings provide important theoretical guidance and technical pathways for developing high-performance SERS substrates.

#### 2.1.3. Research Progress of SERS in Pesticide Detection

SERS has demonstrated broad application potential in the detection of pesticide residues. In the analysis of fruits, vegetables, and milk, high-sensitivity detection has been achieved using SERS substrates such as gold and silver nanoparticles [54,55,56]. By further integrating chemometric methods, researchers have enabled rapid and quantitative detection of various pesticides—including thiabendazole (TBZ) in citrus [57], mixed pesticide residues in apple and orange juices [58], and acetamiprid (ACE), methamidophos, and 2,4-D in tea leaves [59,60].

In the context of staple grains, herbs, and oil-bearing crops, a range of advanced substrates—including Ag@ZnO nanoflowers (NFs), chitosan-modified filter paper, and flower-like AgNPs—have been successfully applied to detect deltamethrin in wheat [61], chlorpyrifos [62], paraquat and thiram in herbal samples [63], and acephate in crude palm oil [64]. These studies consistently highlight the high sensitivity, unique “molecular fingerprint” specificity, and strong adaptability of SERS in complex matrices. Collectively, they underscore SERS as a powerful tool for on-site, rapid screening in food safety monitoring, paving the way for real-time quality control across the agricultural supply chain.

##### SERS Technology in Pesticide Residue Detection in Fruits and Vegetables

SERS has shown tremendous potential in detecting pesticide residues in fruits and vegetables. From citrus to other fruits, vegetable surfaces, fruit juices, and tea leaves, SERS technology offers robust support for on-site rapid detection of food safety issues due to its high sensitivity and unique “molecular fingerprint” characteristics [65,66].

For instance, in the detection of TBZ in citrus, Qin et al. utilized SERS based on gold nanorods (Au NRs) combined with chemometric methods to achieve highly sensitive detection of TBZ residues in citrus. The Au NRs (39 × 22 nm), synthesized via a seed-mediated approach, exhibited a longitudinal plasmon resonance peak at 640 nm and a Zeta potential of +19.8 mV, significantly enhancing the Raman signal. By employing Air-partial least squares (PLS)PLS baseline correction and derivative preprocessing to eliminate interference, and using genetic algorithm-PLS (GA-PLS) to select 144 key variables, the model demonstrated excellent performance (Rp^2^ = 0.9737, RPD = 5.85). This method achieved a detection limit as low as 0.33 μg/mL, far below the Chinese standard (10 mg/kg), with recovery rates of 83.50–98.50% {relative standard deviations (RSD) < 5%} and no significant difference from High-Performance Liquid Chromatography (HPLC) results (*p* > 0.05). Without requiring complex sample preparation, this method can complete detection within 10 min and specifically distinguish coexisting pesticides such as chloropyrifos, providing an efficient and stable solution for pesticide residue detection [57].

Building on these technological advancements, Guo et al. innovatively employed double-layer 4-mercaptobenzoic acid-modified gold-silver core–shell nanoparticles as SERS substrates to achieve efficient dual-mode detection of ACE and benzimidazole in fruits. Through optimized substrate design, the detection limits reached 0.05 mg/kg and 0.03 mg/kg, respectively. This technique demonstrated excellent anti-interference capabilities in actual samples such as apples and pears (recovery rates of 85–110%). Compared to traditional methods, this detection protocol offers advantages such as ease of operation (<10 min), high sensitivity (enhancement factor > 10^6^), and provides reliable technical support for rapid on-site screening of pesticide residues in fruits [67].

**Figure 1:**
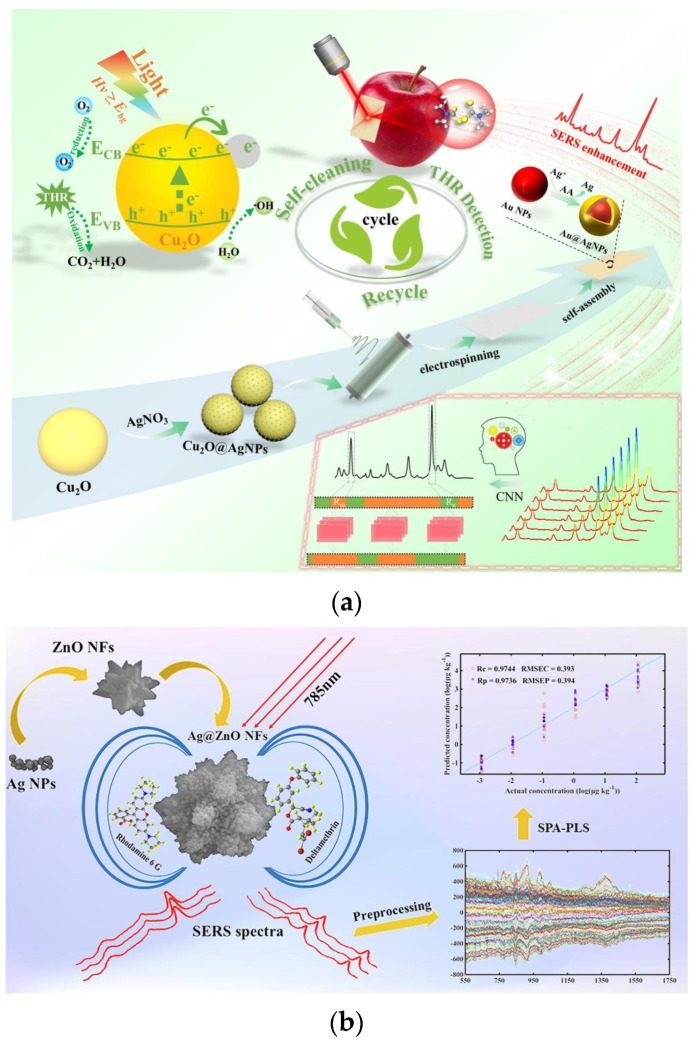
(**a**) PAN/Cu_2_O@Ag/Au@Ag flexible SERS sensor coupled with chemometrics for quantitative detection of thiram residues on apples [68]; (**b**) Quantification of deltamethrin residues in wheat by Ag@ZnO NFs-based SERS coupling chemometric models [61].

In the realm of pesticide detection on fruit surfaces, Guo et al. developed a flexible SERS sensor based on electrospinning and self-assembly of nanomaterials {Polyacrylonitrile (PAN)/Cu_2_O@Ag/Au@Ag} for rapid in situ detection of thiram fungicide on apple surfaces (Figure 1a). The material preparation involved synthesizing PAN/Cu_2_O@Ag flexible substrates via electrospinning and self-assembling Au@Ag core–shell nanoparticles on their surfaces, combining both SERS enhancement and photocatalytic self-cleaning functions. Spectral data were analyzed using convolutional neural networks (CNN) and competitive adaptive reweighted sampling-PLS (CARS-PLS) algorithms, with the CNN model demonstrating superior performance, achieving a correlation coefficient of 0.9963 and a detection limit as low as 0.020 mg/L, below international standard limits. The actual sample detection recovery rates ranged from 88.32% to 111.80%, with the sensor reusable for more than five cycles. When combined with UV photocatalytic degradation, this approach achieved efficient self-cleaning. This technology integrates nanomaterial design with deep learning, offering a highly sensitive and reproducible solution for on-site pesticide residue detection in agricultural products [68].

Notably, for pesticide residues on fruit and vegetable surfaces, Hong et al. innovatively simplified substrate preparation by using commercially available 3 M 9080 tape as a flexible carrier. By optimizing gold nanoparticle (AuNPs) concentration (2.5-fold), dosage (80–120 μL), and sodium chloride coagulant ratio (10–15 μL), they constructed high-activity SERS substrates directly on the tape surface. This method successfully achieved ultra-trace detection of TBZ (20 ng/cm^2^), carbendazim (36 ng/cm^2^), and chlorpyrifos (80 ng/cm^2^) on tomato surfaces. Raman peak assignments were performed using density functional theory (DFT), and semi-quantitative models were established using least squares support vector machines with a Coefficient of Determination (R^2^) ≥ 0.864, significantly enhancing detection accuracy. This strategy simplified the traditional “absorption-separation-dosing” process into a single “adhesion-detection” step. Portable Raman validation showed substrate stability up to 45 days, providing an efficient and practical solution for on-site screening of pesticide residues in agricultural products [69]. While these methods have significantly improved detection convenience and stability, there remains room for further optimization in sensitivity and controllability of hot spot structures. In this context, Li et al. developed a flexible SERS sensor based on electrospinning and electrostatic self-assembly {PDADMAC/Polystyrene sulfonate (PSS)/gold core-silver shell nanorods (Au@Ag NRs) filter paper} for in situ detection of non-systemic pesticides (methyl parathion, thiram, chlorpyrifos) on fruits and vegetables. Cetyltrimethylammonium bromide -guided synthesis of Au@Ag core–shell nanorods was combined with PDADMAC (+) and PSS (−) modification of filter paper to achieve electrostatic self-assembly, forming three-dimensional SERS hotspots. Using 785 nm laser Raman spectroscopy and a “paste-read” method, direct acquisition of epidermal pesticide signals was achieved without complex preprocessing. Detection performance showed limit of detection (LOD) for methyl parathion, thiram, and chlorpyrifos as low as 0.072, 0.052, and 0.059 ng/cm^2^, respectively, with a linear range of 0.051 ng/cm^2^ to 5.096 µg/cm^2^ (R^2^ > 0.988). The actual sample recovery rates were between 64.68% and 126.80%. This sensor offers high sensitivity (LOD for 4-MBA at 10^−12^ M) and stability (RSD < 9.29%), providing a fast (<10 min), non-destructive solution for on-site pesticide residue detection on agricultural products [70].

For rapid and sensitive detection of pesticide residues in complex food matrices like juice, Lin et al. developed a SERS substrate based on vertically aligned gold nanorod arrays (AuNRs) for the detection of the carbamate insecticide carbaryl. Materials were synthesized via seed-mediated growth of the AuNRs with an average length of 87.8 ± 8.9 nm and width of 27 ± 4.6 nm—were synthesized via seed-mediated growth and self-assembled on gold-coated silicon wafers to form vertical arrays. Electromagnetic field enhancement was achieved using 785 nm laser excitation. During detection, samples were centrifuged and directly applied to the substrate without complex preprocessing. The results showed LODs for carbaryl in orange juice, grapefruit juice, and milk at 509, 617, and 391 ppb, respectively, all below US EPA MRLs (juice: 10 ppm, milk: 1 ppm). Prediction models built using PLS showed correlation coefficients (r) between predicted and actual concentrations ranging from 0.88 to 0.95, with recoveries of 82–97.5%. This method offers high sensitivity (minimum detectable level: 50 ppb), speed (sample preparation ~10 min), and good resistance to matrix interference, making it suitable for pesticide residue detection in complex food systems [71].

Building on this, Zou et al. developed a SERS substrate based on Au@Ag NRs for rapid detection of the fungicide TBZ (TBZ) in juices. Materials were synthesized via seed-mediated growth of AuNRs and silver shell deposition to form core–shell structures, with plasmonic resonance optimized by adjusting AgNO_3_ volume (30–250 μL). Uniform nanorods (length: 87.8 ± 8.9 nm, width: 27 ± 4.6 nm) with a silver shell thickness of about 3 nm were obtained. Using 785 nm laser excitation, LODs for TBZ in apple juice and peach juice reached 0.032 ppm and 0.034 ppm, respectively, below EPA limits. Recoveries were 95–101%, with RSD ≤ 4.43%. This method leverages Au@Ag bimetallic synergistic effects (enhancement factor > 10^6^) and vertical adsorption of TBZ molecules’ C-S bonds to the silver shell, significantly enhancing sensitivity. With only 30 min of preprocessing, it is suitable for rapid detection in complex food matrices [72].

To further expand the application of SERS technology in multi-residue detection, Cai et al. utilized portable Raman spectrometers and Au@Ag core–shell nanoparticles SERS substrates to simultaneously detect mixed pesticides (imidacloprid and thiram) in apple juice. By optimizing AgNO_3_ dosage (500 μL/10 mL AuNPs), Au@Ag NPs with optimal SERS activity (~58.6 nm diameter) were obtained. Using 785 nm laser excitation, characteristic peaks at 635 cm^−1^ (imidacloprid C–C–C vibration) and 1385 cm^−1^ (thiram C–N vibration) were selected to build quantitative models. In apple juice matrix, LODs for imidacloprid and thiram were 1.22 μM (0.272 mg/L) and 0.076 μM (0.018 mg/L), respectively, both below EPA MRLs (1 mg/L and 5 mg/L). The recovery rates were 90.2–122.12% and 90.38–113.42%. This method utilizes competitive adsorption mechanisms to distinguish target signals, with the entire detection process taking less than 10 min, significantly improving detection efficiency. It provides an efficient and practical technological pathway for rapid on-site analysis of multiple pesticide residues in agricultural products [58].

Additionally, researchers have extended SERS technology to tea, an important agricultural product, where the complex components, particularly polyphenols, pose significant challenges for detection selectivity and anti-interference performance. To address these challenges, research teams have developed two differentiated detection strategies tailored to tea samples, offering comprehensive solutions for monitoring agricultural product safety.

Chen et al. developed two distinct methods for pesticide detection in tea using SERS technology. Method (i) involves synthesizing 30 nm gold nanoparticles (AuNPs) via sodium citrate reduction, adsorbing crystal violet (CV) as a reporter molecule, and introducing a specific aptamer (ACA) for the insecticide ACE to achieve targeted recognition. When ACA binds with AC, it induces GNP aggregation in salt solutions, forming SERS “hot spots,” thereby enhancing CV’s characteristic peak signal at 1175 cm^−1^. This method exhibits excellent detection performance with a linear range of 3.0 × 10^−8^ to 4.0 × 10^−6^ M and a detection limit as low as 1.76 × 10^−8^ M. The recovery rates in green tea samples were 98.45–104.5%, with RSD less than 5% [59]. Method (ii) optimizes temperature conditions (25 °C) to synthesize flower-like AgNPs with rough surfaces, achieving an enhancement factor of 1.39 × 10^6^ as SERS substrates. Combining solid-phase extraction (SPE) preprocessing techniques, methamidophos, ACE, and 2,4-D were extracted from tea using 80% acetonitrile-water solution, effectively removing matrix interference. SERS detection results showed linear ranges of 1.0 × 10^−3^–10^3^ µg/mL for methamidophos and ACE, and 1.0 × 10^−2^–10^3^ µg/mL for 2,4-D, with LODs of 5.58 × 10^−4^, 1.88 × 10^−4^, and 4.72 × 10^−3^ µg/mL, respectively, all below EU MRLs. The recovery rates were of 84.51–92.58%, with RSD less than 5%, indicating high sensitivity and accuracy. Both methods utilize electromagnetic enhancement mechanisms to identify pesticide characteristic peaks, providing highly sensitive and selective solutions for pesticide residue detection in tea. Method (i) achieves specific recognition through aptamer conformation changes, while method (ii) enhances signals through the rough surface of AgNPs, each offering unique advantages that can be flexibly chosen based on actual detection needs. These studies provide new technological pathways for rapid and accurate detection of pesticide residues in complex matrices such as tea [60].

##### Pesticide Detection in Grains and Oil Crops

SERS has found broad applications in grains and oil crops—an important agricultural sector characterized by complex sample matrix components and diverse pesticide residues, which present significant challenges for analytical detection. To address these challenges, researchers have developed a range of SERS-based detection strategies specifically tailored to the specific the unique properties of individual crop types.

Addressing the issue of deltamethrin residue in wheat, Chen et al. developed a novel SERS detection method based on silver nanoparticle-modified zinc oxide nanoflowers (Ag@ZnO NFs) for the rapid quantitative analysis of deltamethrin residues in wheat (Figure 1b). The Ag@ZnO NFs, synthesized via wet chemistry methods, feature a three-dimensional structure with an enhancement factor reaching 10^7^, effectively boosting the Raman signal of target molecules. After ethanol extraction of samples, mean centering (MC) preprocessing and successive projections algorithm-PLS regression (SPA-PLS) modeling were employed, achieving a detection limit of 0.16 μg/kg, with a linear range of 1.0 × 10^−3^ to 10^2^ μg/kg. The spiked recovery rates ranged from 96.33% to 109.17% (RSD < 5%). By optimizing substrate morphology and chemometric models, this method significantly enhanced detection sensitivity and accuracy, providing an efficient solution for monitoring pesticide residues in agricultural products [61].

Notably, researchers have developed differentiated detection strategies for various types of pesticide residues in wheat. For instance, to detect chlorpyrifos, an organophosphate pesticide, Huang et al. utilized more affinity-based substrate materials combined with SERS to achieve highly sensitive detection of its residues in wheat. Using wet chemistry synthesis of Ag@ZnO nanoflowers (Ag@ZnO NFs), their three-dimensional structure and LSPR effects reduced the detection limit of deltamethrin to 0.16 μg/kg, with recovery rates ranging from 96.33% to 109.17%. The use of MC-SPA-PLS models optimized prediction performance {Prediction Correlation Coefficient (Rp) = 0.9736}. Another study used chitosan-modified filter paper as a substrate, optimizing acetic acid concentration (1.5% optimal) to enhance the uniform distribution of AgNPs, achieving a chlorpyrifos detection limit of 0.000558 mg/L. Normalized PLS model Rp reached 0.9764, with recovery rates of 97.25–119.38% [62].

Shifting focus from solid grain crops to liquid oil detection, SERS technology demonstrates remarkable adaptability and detection capabilities. In the highly complex matrix of crude palm oil, SERS technology excels particularly well. Addressing acephate (ACE) residue issues in crude palm oil, Li et al. employed flower-like AgNPs (AgNPs) as substrates, combined with the random frog (RF) algorithm, to achieve high-sensitivity precise detection. This method achieved a detection limit as low as 4.69 ng/g, exhibited excellent linearity (R^2^ > 0.99), and demonstrated high recovery rates (93.89–108.32%), fully demonstrating the robust capability of SERS technology in liquid oil detection [64].

SERS technology exhibits distinct performance characteristics across different matrices in pesticide residue detection. For fruits and vegetables, where residues are typically surface-localized, flexible substrates (e.g., tape- or electrospun membrane-based sensors) enable direct “stick-and-read” detection with high sensitivity (LODs down to 0.05 ng/cm^2^) and minimal sample preparation, offering excellent potential for on-site screening—though surface heterogeneity can compromise reproducibility. In liquid matrices such as juices, simplified workflows involving centrifugation followed by direct drop-casting on vertically aligned AuNRs or Au@Ag NRs substrates achieve ppb-level sensitivity (e.g., 0.032 ppm for TBZ). However, matrix components like pigments and organic acids may interfere with target adsorption, necessitating chemometric correction. In contrast, grain matrices require solvent extraction (e.g., acetonitrile/water) due to the deep embedding of pesticides within complex carbohydrates, and substrates like Ag@ZnO nanoflowers provide strong signal enhancement (up to 10^7^) and low detection limits (e.g., 0.16 μg/kg for deltamethrin), albeit at the cost of longer processing times and reduced field applicability. For oily matrices such as crude palm oil, high viscosity and intense fluorescence severely suppress Raman signals, posing significant challenges for uniform substrate dispersion and signal stability—even with flower-like AgNPs, despite achieving low LODs (e.g., 4.69 ng/g for acephate). Overall, SERS performs most effectively in surface and liquid systems for rapid screening, while its application in lipid-rich or solid-dense matrices demands more sophisticated sample preparation and signal enrichment to overcome strong matrix interference.

##### Application of Chemometrics Combined with SERS Technology in Pesticide Detection

Data processing in SERS has evolved into an innovative, multi-technology analytical framework. In the field of chemometrics, PCA enables efficient data compression through feature space reconstruction, PLS establishes precise quantitative relationships between spectral signals and analyte concentrations, while the RF algorithm significantly enhances model generalization by intelligently selecting key spectral variables [73,74]. Emerging deep learning techniques are complementing traditional methods: CNNs can automatically extract complex spectral features, while transfer learning effectively addresses the challenge of limited training data in real-world applications [75]. The synergistic application of these approaches—such as combining PCA-based preprocessing with deep learning models—retains the interpretability of chemometric methods while fully exploiting the nonlinear modeling power of advanced algorithms. This hybrid strategy enhances both accuracy and robustness in SERS-based detection, particularly in complex matrices.

This synergistic effect is particularly evident in the detection of pesticide residues in tea. Chen et al. developed an innovative analytical platform that integrates SERS with chemometric modeling for the detection of chlorpyrifos residues in tea. The team designed and synthesized core–shell Au@Ag nanoparticles as SERS substrates, which exhibited a high enhancement factor of 2.5 × 10^6^. For qualitative analysis, a K-Nearest Neighbors algorithm combined with second-derivative spectral preprocessing achieved highly accurate classification of chlorpyrifos-contaminated tea samples, with classification accuracy ranging from 90.84% to 100%. In quantitative analysis, GA-optimized models—GA-PLS and siPLS-GA—were applied to Standard Normal Variate (SNV)-preprocessed spectra, yielding excellent predictive performance: determination coefficients (r^2^) reached 0.96–0.98, and root mean square errors (RMSE) of prediction (RMSEP) were maintained between 0.29 and 0.31. No statistically significant difference was observed between the results of this method and reference GC-MS measurements (*p* > 0.05), and the detection limit was as low as 3.0 × 10^−9^ mol/L. This integrated approach provides a rapid and highly sensitive tool for quality control and safety monitoring in the tea industry [76].

Notably, Chen et al. further advanced this methodology by combining octahedral hollow Au-Ag cages (Au-Ag OHCs) with a CNN algorithm. The unique plasmonic structure of the Au-Ag OHCs provided strong electromagnetic enhancement, while the CNN enabled intelligent feature extraction from complex spectral data, pushing detection sensitivity into the parts-per-billion (ppb) range. By synergistically applying CNN, PLS, and Extreme Learning Machine (ELM) algorithms, the method achieved accurate quantitative prediction of thiram and pymetrozine residues in tea. Statistical comparison with HPLC reference data revealed no significant difference (*p* > 0.05), confirming the reliability and accuracy of the SERS-chemometrics framework [77].

##### Application of Molecularly Imprinted Polymers (MIPs) with SERS Technology in Pesticide Detection

SERS exhibits highly sensitive, yet it is prone to interference from co-existing substances within complex matrices, thereby limiting its practical applications. To tackle this challenge, researchers have integrated MIPs—which are known for them possess specific recognition capabilities—with SERS technology. This resultant combination has facilitated the creation of innovative sensing platforms that deliver both enhanced sensitivity and specificity, thereby offering a solid foundation for the swift and precise detection of pesticide residues in foodstuffs [78,79].

**Figure 2:**
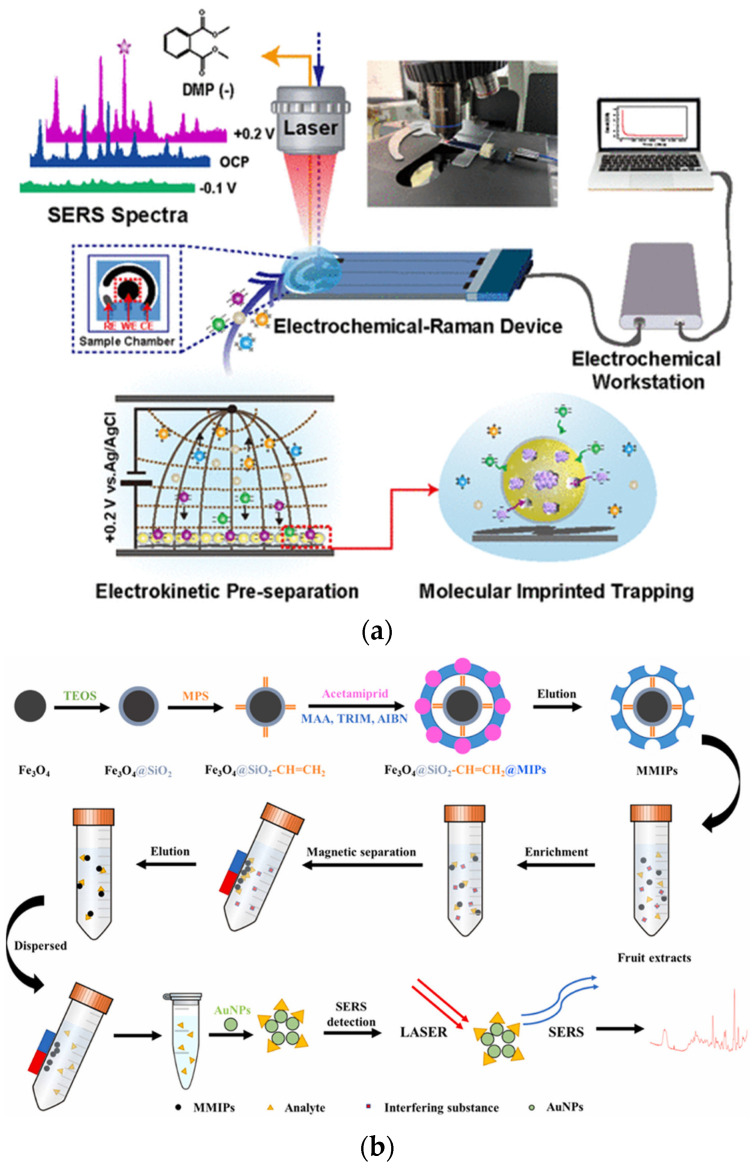
(**a**) Illustration of Electrokinetic Preseparation and Molecularly Imprinted Trapping of Charged PAEs on a Portable Interface for Selective SERS Recognition [80]; (**b**) Detection of neonicotinoids in agricultural products using magnetic MIPs-surface enhanced Raman spectroscopy [81].

MIPs are typically synthesized using target pesticides or their structural analogs as template molecules. These templates interact with functional monomers (e.g., methacrylic acid, acrylamide) and cross-linkers {e.g., ethylene glycol dimethacrylate (EGDMA)} to form specific recognition cavities. The resulting MIPs are then integrated with SERS substrates such as gold/AgNPs or magnetic Fe_3_O_4_@SiO_2_ composite materials (Figure 2a), forming either “one-step” or “two-step” sensors [80].

For the detection of lipophilic pesticides, traditional aqueous-phase SERS systems are not suitable. Neng et al. developed MIPs using methyl methacrylate as the functional monomer and pentachloronitrobenzene (PCNB) as the template molecule, integrating oil-soluble AgNPs as the SERS-active substrate. The prepared MIPs exhibited specific recognition capability towards PCNB, with an adsorption equilibrium time of only 120 min. Using the optimized SERS-MIPs method, a good linear relationship was observed within the concentration range of 0.005–0.15 μg/mL, with a detection limit of 5.0 ng/mL. In spiked rice samples, the recovery rates were between 94.4% and 103.3%, with RSD ranging from 4.6% to 7.4%, consistent with GC-MS results. This method combines the selective enrichment capability of MIPs with the high sensitivity of SERS, offering a rapid and reliable solution for detecting lipophilic pesticide residues in food matrices [82].

In the realm of detecting hydrophilic and polar pesticides, researchers have significantly improved the performance of SERS-MIPs sensors through the optimization of functional monomers and substrate structures. Yan et al. developed MIPs integrated with AuNPs) combined with SERS technology for simultaneous detection of triazine herbicides prometryn and simetryn. Through precipitation polymerization, MIPs with specific-like recognition properties were synthesized, exhibiting imprinting factors of 5.3 and 4.2 (at an initial concentration of 10 μg/mL) for prometryn and simetryn, respectively, with an adsorption equilibrium time of only 60 min. Optimized AuNPs (~50 nm diameter) served as the SERS substrate, with NaCl used as an aggregating agent. Quantitative methods were established at characteristic peaks of 974 cm^−1^ (prometryn) and 1074 cm^−1^ (simetryn). The method demonstrated detection limits of 20 μg/kg in rice and wheat samples, with a linear range of 0.02–0.5 μg/mL. The recovery rates ranged from 72.7% to 90.9% (RSD: 1.7–7.8%), and effective elimination of matrix interference in grain samples was achieved through the combination of MIPs with QuEChERS (Quick, Easy, Cheap, Effective, Rugged, and Safe), enabling highly selective detection of dual residues in complex samples [83].

Cao et al. developed a novel detection method based on magnetic MIPs coupled with SERS (MMIPs-SERS) (Figure 2b) for the rapid analysis of neonicotinoid pesticides (ACE and thiacloprid) in agricultural products. By employing surface imprinting techniques to polymerize an imprinting layer on the surface of magnetic nanoparticles, they created core–shell structured MMIPs that achieve adsorption saturation within just one minute and exhibit specific-like recognition capabilities towards the target analytes. The optimized MMIPs-SERS system demonstrated a linear detection range of 1–20 μg/g for ACE and thiacloprid in pear and peach samples, with detection limits of 23.7–68.8 ng/g and 23.7–36.4 ng/g, respectively. The spiked recovery rates ranged from 73.5% to 112.8%, with (RSD below 7.0%, validating the high sensitivity and accuracy of this method. This approach leverages the selective enrichment capability of MMIPs and the rapid analysis advantages of SERS, providing an efficient solution for detecting trace pesticide residues in complex matrices [81].

Xu et al. reported a highly sensitive SERS sensor based on magnetic MIPs (Mag@MIP NPs) combined with gold Au NPs for the detection of 2,4-dichlorophenoxyacetic acid (2,4-D) in food and water. Magnetic nanoparticles (200 nm diameter) were synthesized using FeCl_3_·6H_2_O and chitosan, and an imprinting layer was formed by polymerizing acrylamide functional monomers and EGDMA cross-linkers in the presence of 2,4-D template molecules, resulting in a 14 nm thick imprinting layer. The Mag@MIP NPs specifically captured 2,4-D via hydrogen bonding, achieving adsorption equilibrium within 120 min. Subsequently, the SERS signal was enhanced through electrostatic adsorption onto Au NPs, with characteristic peaks at 1071 cm^−1^. The sensor exhibited a linear response in the concentration range of 0.1–10^5^ ng/mL (R^2^ = 0.988), with a detection limit as low as 0.00147 ng/mL. The spiked recovery rates for milk and tap water samples were between 93.5% and 102.2%, showing no significant difference compared to HPLC results (*p* > 0.05). This demonstrates the excellent selectivity and practicality of the sensor [84].

To further enhance sensor performance, some studies focus on the synergistic optimization of functional monomers and substrate morphology. For instance, Li et al. developed a self-cleaning R6G detection sensor using zinc oxide nanorods as the substrate and AgNPs-modified ZnO/Ag heterostructures (ZOA) as the SERS-active substrate, combined with molecular imprinting technology. During the preparation process, acrylamide was used as the functional monomer and ethylene glycol dimethacrylate as the cross-linker to construct R6G-specific recognition cavities on the ZOA surface, achieving an imprinting factor of 5.3. The optimized ZOA-MIPs exhibited excellent selectivity at the characteristic peak of 1654 cm^−1^, with a detection limit of 10^−13^ mol/L (R^2^ = 0.996) and an enhancement factor of 3.3 × 10^5^. Cross-reactivity with structurally similar compounds such as rhodamine B and CV was below 30%. Under UV light irradiation, the sensor could achieve self-cleaning through photocatalysis, maintaining over 92% signal stability after five cycles of use. This demonstrates high sensitivity, selectivity, and reproducibility [85].

Additionally, the “two-step” strategy offers new approaches for detecting pesticide residues in complex matrices. Hua et al. synthesized specific MIPs targeting 2,4-D and innovatively combined them with AgNPs-modified SERS substrates. Using molecularly imprinted SPE (MISPE) technology, they selectively enriched 2,4-D from milk samples, establishing a highly sensitive detection method at the characteristic peak of 391 cm^−1^. This method demonstrated excellent analytical performance: a detection limit as low as 0.006 ppm, a quantification limit of 0.008 ppm, and a good logarithmic linear relationship within the concentration range of 0.01–1 ppm (R^2^ = 0.9887), fully covering the MRLs set by European and American regulations for 2,4-D in milk (0.01–0.05 ppm). By optimizing the QuEChERS pretreatment combined with MISPE technology, the spiked recovery rates for milk samples were stable between 85% and 95%, with RSD below 8%. The entire detection process took only 20 min, providing a reliable technique for rapid screening of pesticide residues in dairy products [86].

The integration of SERS with MIPs has shown considerable promise for pesticide residue detection. Through rational design of functional monomers, optimization of substrate architectures, incorporation of functionalities such as magnetic separation or self-cleaning, and implementation of “two-step” enrichment strategies, this hybrid approach has enabled highly sensitive and selective detection of a wide range of pesticides—from lipophilic and hydrophilic species to analytes in complex matrices. Future research could focus on expanding the multi-residue recognition capability of MIPs, improving the stability and reproducibility of SERS substrates, and advancing the practical implementation of this technology in on-site, rapid screening for food safety monitoring.

In summary, these technological innovations have collectively driven a leap forward in SERS-based detection. Continuous advancements in substrate materials, coupled with algorithmic optimization, have enabled SERS to overcome the longstanding challenges of detecting trace-level pesticides in complex food matrices. The technique’s unique molecular fingerprinting capability ensures accurate target identification, while diversified substrate designs enable multiple pathways for signal enhancement. The synergistic use of chemometric and deep learning algorithms not only significantly improves detection accuracy but also establishes a complete technical pipeline—from measurement and analysis to data processing [87]. These breakthroughs offer rapid, non-destructive solutions for food safety monitoring and hold great promise for large-scale industrial applications.

#### 2.1.4. Comparison of Various SERS Detection Methods

Table 1 provides a systematic comparison of multiple SERS detection methods used in pesticide residue analysis, highlighting significant advancements in sensitivity, accuracy, and practicality. Overall, various SERS methods generally exhibit high detection sensitivity, with LOD ranging from ppm to ppb levels, and even down to ng/cm^2^ or ng/g. Some advanced strategies demonstrate exceptional trace detection capabilities; for instance, the LOD for 2,4-D reaches as low as 0.00147 ng/mL [84], while the integration of CNN models achieves an LOD of thiram as low as 0.286 ppb [77]. These examples underscore the immense potential of combining SERS with intelligent algorithms for signal resolution and sensitivity enhancement.

In terms of detection accuracy, most methods achieve recovery rates within the ideal range of 80–120%. For example, the recovery rates for ACE and cypermethrin are 93.86–105.64% and 92.62–102.3%, respectively [67], indicating good quantitative reliability. Additionally, RSD are generally low (mostly <10%). For instance, the RSD for thiram detection is 2.29% [54], demonstrating excellent repeatability and stability of the results.

The widespread use of portable Raman spectrometers has significantly reduced detection times to within 5–30 min, greatly enhancing the feasibility of on-site rapid screening [54,56,72,81]. Further improvements in detection performance have been achieved by incorporating chemometrics, MIPs, or core–shell nanomaterials. For example, MIPs-SERS achieves an LOD of 0.005 µg/mL for pentachloronitrobenzene (PCNB) with stable recovery rates of 94.4–103.3% [82], while SERS combined with chemometrics achieves an LOD of 0.16 μg/kg for deltamethrin [61]. Although confocal Raman microscopy may extend detection times (e.g., up to 2 h) [84], it enables ultra-trace analysis with extremely high precision.

In summary, SERS exhibits strong potential for pesticide residue detection, owing to its high sensitivity, rapid response, and good reproducibility [53]. When combined with novel materials, advanced algorithms, and portable devices, SERS approaches increasingly meet the comprehensive needs of efficient, precise, and on-site detection in real-world scenarios. This integration not only enhances selectivity toward target analytes but also significantly improves overall detection performance, establishing SERS as a highly valuable tool for food safety monitoring.

### 2.2. Infrared Spectroscopy

#### 2.2.1. Principles

At the heart of infrared (IR) spectroscopy is the interaction between the vibrational energy levels of molecules and infrared radiation. When the frequency of incoming light aligns with the vibrational frequency of a molecule’s chemical bonds, the molecule selectively absorbs specific wavelengths of energy due to changes in its dipole moment, leading to distinctive absorption bands [88]. This technique can be divided into three spectral regions based on wavelength: the near-infrared region (0.75–2.5 μm), which mainly reflects overtones of molecular vibrations; the mid-infrared region (2.5–25 μm), which contains rich information about fundamental vibrational modes; and the far-infrared region (25–1000 μm), which involves changes in molecular rotational energy levels [89]. Recent studies highlight that mid-infrared spectroscopy, owing to its enhanced resolution and specificity, has emerged as the predominant technique for structural elucidation of materials [90]. However, when applying IR spectroscopy to trace analytes, it is limited by low infrared absorption cross-sections. Surface-enhanced infrared absorption spectroscopy (SEIRAS), an advanced form of IR spectroscopy, overcomes this limitation [91].

In the field of pesticide residue detection, infrared spectroscopy exhibits unique advantages. NIR spectroscopy, with its rapid detection capabilities, is suitable for preliminary screening of active ingredients such as deltamethrin in pesticide formulations. However, its analysis results are constrained by interference from water and model stability issues [92]. In contrast, mid-infrared spectroscopy can establish more precise quantitative models by leveraging characteristic absorptions of functional groups like C=O and C-N in pesticides such as imidacloprid, combined with PLS regression algorithms. Additionally, SEIRAS technology can overcome the limitations of sensitivity and precision inherent in conventional mid-infrared spectroscopy [93].

#### 2.2.2. Research Progress of Infrared Spectroscopy in Pesticide Detection

With the rapid development of infrared spectroscopy technology, its application in the detection of dithiocarbamate (DMD) pesticides has gradually gained attention. Alessandra et al. developed a method based on flow injection-Fourier transform infrared spectroscopy for the determination of ziram and thiram in solid samples. This study involved loading AgNPs (10–100 nm in diameter) onto a copper foam substrate via a displacement reaction method. The process was optimized with parameters such as silver nitrate concentration (0.2 mmol/L), polyvinylpyrrolidone (PVP) amount (2 mL), and reaction time (30 s) to successfully prepare a transmission surface-enhanced infrared absorption spectroscopy (T-SEIRAS) active substrate. Scanning electron microscopy (SEM) and X-ray photoelectron spectroscopy characterization confirmed that the AgNPs were uniformly distributed on the substrate surface. This substrate achieved a 32.7-fold enhancement in the infrared absorption signal of the probe molecule Mercaptoundecanoic Acid at 1689 cm^−1^ and a 2.9-fold enhancement for thiram at 1371 cm^−1^. By constructing a linear response model for thiram at 1236 cm^−1^ (R^2^ = 0.923), the detection limit reached as low as 0.024 mg/mL. This method offers advantages such as fast detection (approximately 5 min) and low cost, providing new insights for on-site rapid pesticide residue detection [94].

Notably, breakthroughs in Surface-Enhanced Infrared Absorption (SEIRA) technology not only offer new methods for detecting specific pesticides like thiram but also enhance overall detection capabilities through innovations in substrate materials. This material-driven technological advancement is driving pesticide residue detection toward higher sensitivity and broader applicability.

The latest breakthroughs in SEIRA technology for pesticide residue detection are mainly reflected in three aspects: (1) Innovations in Substrate Materials: Researchers have developed novel noble metal nanostructures (such as gold and AgNPs) and cutting-edge nanomaterials like graphene and hybrid perovskites, creating high-performance SEIRA substrates. These material innovations have significantly improved infrared signal sensitivity by up to three orders of magnitude, with enhancement factors reaching 10^3^–10^4^ [95]. (2) Optimization of Detection Methods: Improvements in spectral acquisition parameters and signal processing algorithms have further enhanced detection accuracy and stability. (3) Expansion of Application Scenarios: The technology has been successfully applied to new areas such as complex matrix detection and on-site rapid screening. This triad of material innovation, method optimization, and expanded applications is driving pesticide residue detection technology toward higher sensitivity and stronger interference resistance.

##### High-Performance SEIRA Enabled by Metallic Nanostructures

**Figure 3:**
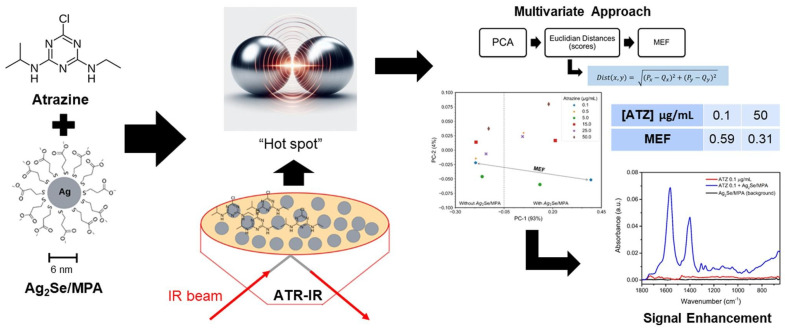
A SEIRA multivariate approach for ATZ detection [96].

Pereira et al. developed a green analytical method for detecting atrazine (ATZ) by combining SEIRA with silver selenide quantum dots (Ag_2_Se/MPA) (Figure 3). The Ag_2_Se/MPA quantum dots (6 ± 3 nm in size) were synthesized via a one-pot method and used as the SEIRA-active substrate. Coupled with attenuated total reflection infrared spectroscopy (ATR-IR), the method leverages electrostatic interactions and electromagnetic enhancement between the quantum dots and ATZ molecules to achieve an 86-fold signal enhancement at 1547 cm^−1^ (νC=N stretching vibration). The method demonstrated a low detection limit of 0.001 µg/mL (1 ng/mL) and a linear range of 0.1–50 µg/mL. Validation using a PLS regression (PLSR) model showed recovery rates consistent with those obtained by chromatographic methods while eliminating the need for the complex sample preparation typically required in conventional chromatography. This SEIRA-based strategy significantly enhances the sensitivity of infrared detection, meeting the regulatory limits for ATZ in water set by Brazil and the European Union (2 µg/L). It offers a rapid, cost-effective, and environmentally friendly solution for monitoring environmental pollutants [96].

In the development of SEIRA substrates based on metallic nanomaterials, Gong et al. achieved a significant advance in pesticide detection through the fabricating of a silver nanoparticle-coated copper foam (AgNP/Cu foam) substrate—a rationally designed metallic nanostructure that dramatically enhances the infrared signals of target molecules. In the detection of thiram, this approach achieved a limit of detection (LOD) as low as 0.024 mg/mL, representing an improvement of two orders of magnitude compared to conventional infrared spectroscopy. Furthermore, the method enables precise identification of molecular fingerprint features, such as the characteristic absorption peak at 1236 cm^−1^.

The innovation lies in its transmission-mode SEIRA (T-SEIRA) configuration combined with a three-dimensional porous substrate design. This architecture ingeniously overcomes the inherent limitation of poor transparency in metallic materials, enabling rapid, in situ detection without complex sample pretreatment. In contrast, traditional infrared spectroscopy is severely constrained by background interference at low concentrations and cumbersome sample preparation procedures [97]. These distinct advantages clearly highlight the transformative potential of SEIRA technology in the field of pesticide analysis.

##### SEIRA Substrates Based on Hybrid Perovskites

In recent years, hybrid organic-inorganic perovskites have emerged as promising materials in optoelectronics and sensing due to their excellent photoelectric properties and tunable bandgaps (1.3–1.7 eV) [98]. Their unique semiconductor characteristics—such as high charge carrier mobility and strong plasmonic effects—offer a new class of low-cost, highly sensitive substrates for SEIRA [99].

Studies have demonstrated that, through rational design and optimization, perovskite-based substrates exhibit outstanding performance in pesticide residue detection, advancing the capabilities of trace-level analytical techniques. In SEIRA applications, CH_3_NH_3_PbX_3_ (X = Cl, Br, I) thin films have been innovatively employed as enhancing substrates. These uniform and stable films are fabricated via solvent evaporation or spin-coating followed by annealing (100 °C for 45 min). Characterization results confirm their good thermal stability and typical semiconductor behavior. The synergistic effect of surface plasmon resonance and carrier dynamics significantly enhances the infrared signals of target molecules. Comparative experiments reveal that CH_3_NH_3_PbBr_3_ achieves a signal enhancement factor of up to 150 for acrylamide, outperforming CH_3_NH_3_PbCl_3_ and CH_3_NH_3_PbI_3_, indicating a strong influence of halide composition on enhancement performance. These findings highlights that the band structure and charge carrier dynamics of perovskite materials are critical factors governing SEIRA efficiency [99].

Leveraging these advantages, researchers have successfully applied CH_3_NH_3_PbBr_3_-based substrates to the detection of pesticide residues in real samples. Using benzoyl peroxide (BPO)—a common additive in flour—as a model analyte, high-sensitivity detection was achieved at the characteristic peaks of 1759 cm^−1^ and 1224 cm^−1^. The method exhibited a linear detection range of 0.004–0.064 mol/L with correlation coefficients (R^2^) of 0.9958 and 0.9975, respectively, demonstrating excellent quantitative capability. More importantly, the recovery rates in real samples reached 100.4–101.0%, with RSD no greater than 1.42%, indicating high accuracy and reproducibility. The substrate can be regenerated via a simple acetone rinse and reused multiple times, substantially reducing operational costs. This approach offers a highly efficient and cost-effective solution for food safety monitoring [99].

To further improve detection limits and material stability, Wang et al. developed a core–shell nanostructure based on CdS-coated CsPbX_3_ (X = Cl, Br) quantum dots (CPCBM/CdS) [100]. This design effectively addresses the inherent instability of traditional perovskites in aqueous environments, greatly enhancing durability. By introducing Mn/Br co-doping to induce spin polarization and applying external magnetic field regulation, the carrier lifetime was extended to 4244 ps—approximately 2.2 times longer than in the unmodulated system. Based on this enhanced material, a photoelectrochemical sensor was constructed by integrating microfluidic paper-based analytical devices (μ-PADs) and a DNA nanowire-based signal amplification strategy. This platform enabled ultra-sensitive detection of the neonicotinoid pesticide ACE. The method achieved an exceptionally low detection limit of 23 fM, with a linear range spanning five orders of magnitude (10^−13.5^ to 10^−9^ M). In practical tests on agricultural products such as tomatoes and cucumbers, the recovery rates ranged from 90.7% to 105.6%, with RSD below 5.29%, demonstrating outstanding reliability and applicability.

Perovskite-based SEIRA outperforms silver nanostructure-based methods in sensitivity and quantitative reliability under optimized conditions. While Ag_2_Se quantum dots achieve a 1 ng/mL LOD for ATZ—meeting EU water standards (0.1–0.2 µg/L)—and AgNP/Cu foam detects thiram at 24 µg/mL, perovskite systems such as CsPbX_3_/CdS enable ultra-trace detection of ACE down to 23 fM (~7 × 10^−3^ µg/L), exceeding EU MRLs in crops by several orders of magnitude [96,97,100]. This superior performance stems from enhanced carrier dynamics and plasmonic effects in perovskites, particularly under magnetic regulation, which prolong carrier lifetime and boost signal amplification. Furthermore, CH_3_NH_3_PbBr_3_ substrates show excellent linearity (R^2^ > 0.995) and near-ideal recovery rates (100.4–101.0%) [99], well within the EU’s acceptance criteria (70–120% recovery, RSD < 15%), demonstrating high accuracy and suitability for regulatory analysis. Even in complex food matrices, perovskite-based detection maintains recoveries of 90.7–105.6% with RSD < 5.3%, comparable to chromatographic reference methods. Additionally, their reusability and low fabrication cost enhance practicality for routine monitoring. Therefore, in applications requiring ultra-sensitivity, compliance with stringent regulations, and cost-effective repeated use—such as food safety screening—perovskite-based SEIRA offers greater analytical advantage and real-world applicability than metallic nanostructure-based approaches [95].

In summary, hybrid perovskites have become ideal candidates for SEIRA substrate development, thanks to their tunable optoelectronic properties and significant plasmonic enhancement effects. From thin-film substrates to core–shell quantum dots, and from single enhancement mechanisms to multi-strategy synergistic designs, their application in pesticide residue detection continues to deepen and evolve. Future advancements through material engineering, surface functionalization, and device integration are expected to enable perovskite-based sensing platforms to achieve even higher sensitivity, selectivity, and broader applicability for on-site, rapid detection. These developments hold strong promise for providing robust technological support in food safety and environmental monitoring.

##### Methodological Innovations and Hyphenated Techniques in SEIRA

In recent years, methodological advancements and the integration of complementary technologies have become key drivers in the development of SEIRA. Among these, the coupling of SEIRA with electrochemistry (EC-SEIRAS) and the integration of porous materials have emerged as prominent research frontiers.

EC-SEIRAS systems enable in situ monitoring of pesticide degradation pathways, offering real-time molecular-level insights into reaction mechanisms. Meanwhile, the incorporation of three-dimensional porous substrates—such as filter paper or copper foam—combined with physical filtration techniques, allows for the simultaneous enrichment and detection of pesticide residues. This synergistic approach significantly enhances sensitivity and simplifies sample handling, particularly for trace analysis in complex matrices [101].

For DMD pesticides, Gong et al. demonstrated that an optimized silver nanoparticle-coated copper foam substrate achieved a 2.9-fold enhancement for thiram at 1371 cm^−1^, while a gold nanoparticle-based substrate exhibited an even more remarkable 188.2-fold enhancement for ziram [102]. These results highlight the critical role of metal type and nanostructure design in maximizing SEIRA performance.

Despite significant advances, several challenges persist. Enhancing substrate stability under operational conditions and mitigating interference from complex sample matrices—such as those food in food and environmental samples—remain critical barriers to real-world deployment. Emerging materials such as phonon polariton resonators—known for their low optical losses and narrow resonance peaks—hold promise for pushing the sensitivity limits of SEIRA to new levels. Moreover, AI-driven spectral analysis algorithms are expected to play a transformative role in optimizing quantitative models. Techniques such as PLSR could be significantly enhanced through machine learning-assisted peak deconvolution, baseline correction, and multivariate calibration, leading to more robust and accurate detection.

Current research indicates that SEIRA technology is transitioning from laboratory-based studies toward field-deployable, rapid detection systems. With ongoing advances in material design, method integration, and data analytics, SEIRA is evolving into a powerful, portable, and efficient solution for pesticide residue monitoring—offering strong support for regulatory compliance and public health protection.

##### SWOT Analysis: Surface-Enhanced Techniques in Raman and IR Spectroscopy for Pesticide Detection

Surface-enhanced techniques, particularly SERS and SEIRA, offer exceptional sensitivity in pesticide detection. SERS can amplify Raman signals by up to 10^14^ times, enabling trace-level (ppb to ppt) detection, while SEIRA enhances infrared absorption by 10^2^–10^4^ times. Both techniques preserve the unique vibrational “fingerprint” of pesticide molecules, allowing for specific identification. Their rapid, non-destructive nature and compatibility with minimal sample preparation make them highly suitable for on-site screening in food safety and environmental monitoring.

Despite their high sensitivity, these techniques face significant challenges related to substrate performance. The fabrication of reproducible, uniform, and stable SERS/SEIRA substrates—often based on noble metal nanostructures—is complex and costly. Signal reproducibility across batches remains a major hurdle for reliable quantitative analysis. Additionally, the stability of substrates (especially silver-based ones) is limited due to oxidation or sulfidation. The interaction between the pesticide and the substrate surface can also influence spectral features, complicating data interpretation and affecting measurement accuracy.

Emerging opportunities lie in the development of novel nanomaterials—such as 2D materials, MOFs, and hybrid nanostructures—that offer improved stability, tunability, and lower cost. Functionalizing substrates with recognition elements (e.g., aptamers, molecularly imprinted polymers) can enhance selectivity for specific pesticides. The integration of SERS/SEIRA with portable spectrometers enables the creation of handheld devices for real-time field analysis. Furthermore, combining these techniques with machine learning for spectral analysis and exploring multimodal detection strategies can significantly improve reliability and automation.

The widespread adoption of surface-enhanced techniques is challenged by well-established analytical methods like GC-MS and LC-MS, which offer high accuracy, multi-residue capability, and standardized protocols. The high cost and difficulty of mass-producing high-performance substrates limit commercial scalability. Complex sample matrices (e.g., fruits, soil) can introduce interference, reducing detection reliability.

In summary, SERS and SEIRA hold transformative potential for pesticide detection due to their unparalleled sensitivity and molecular specificity. While challenges in substrate reproducibility, stability, and standardization remain critical weaknesses, ongoing advancements in nanotechnology and device integration present significant opportunities [101]. To overcome existing threats from conventional methods and gain broader acceptance, future efforts should focus on developing robust, low-cost substrates, establishing standardized protocols, and validating performance in real-world scenarios. With continued innovation, surface-enhanced spectroscopic techniques are poised to become powerful tools for rapid, on-site pesticide residue analysis.

### 2.3. Fluorescence Spectroscopy

#### 2.3.1. Principle

Fluorescence spectroscopy is an analytical method that measures the characteristic fluorescence emission from pesticide molecules upon excitation at a specific wavelength. The principle involves molecules absorbing light energy, causing electronic transitions, and emitting fluorescence at a shifted wavelength as they return to the ground state. Quantitative detection is achieved by analyzing the fluorescence intensity and characteristic spectral lines [103,104]. This technique is noted for its simplicity, speed, high sensitivity, selectivity, and ability to provide extensive molecular information about the substances being tested, making it particularly suitable for detecting trace substances.

The technology is primarily divided into three types based on spectral type: front-face fluorescence spectroscopy (for direct detection of solid sample surfaces), synchronous fluorescence spectroscopy (simultaneously scanning excitation and emission wavelengths), and three-dimensional fluorescence spectroscopy {providing excitation-emission matrix (EEM) information}. Three-dimensional fluorescence can acquire richer molecular structure data. In terms of detection methods, there are three categories: direct detection is suitable for naturally fluorescent pesticides like carbendazim, with detection limits reaching 0.005 mg/kg; derivatization enhances non-fluorescent pesticide signals through chemical modifications, common markers include dansyl chloride and quantum dots; probe methods utilize molecular recognition mechanisms such as aptamers and imprinted polymers for specific sensing. The advantages include ultra-high sensitivity (2–3 orders of magnitude higher than chromatography), rapid detection (<3 min), and a trend towards portability. However, it has three main limitations: only 35% of pesticides have intrinsic fluorescence; matrix interference results in a false positive rate as high as 22%; and it cannot detect systemic pesticide residues [105]. Recent advancements involve time-resolved fluorescence (with lifetime differences >10^6^ folds) to eliminate background interference, and the combination of three-dimensional fluorescence with chemometrics for simultaneous analysis of multiple components [106].

Research has shown that conventional fluorescence detection relies on Stokes shift—where the emission wavelength longer than excitation wavelength—whereas emerging fluorescence systems employ upconversion and ratiometric fluorescence modes by modulating the interactions between luminescent materials and targets [107,108]. Upconversion fluorescence employs rare-earth doped nanoparticles (e.g., NaYF_4_:Yb^3+^, Er^3+^) with multiphoton absorption characteristics to convert NIR light (like 980 nm) into visible light emissions, effectively avoiding interference from autofluorescence of biological tissues [109]. Additionally, upconversion fluorescence probes, through surface modifications with aptamers or MIPs, can specifically recognize organophosphorus pesticides (OPPs) [110]. Ratiometric fluorescence constructs dual-emission probes (e.g., silicon-carbon dots combined with gold nanoclusters) using the intensity ratio of two fluorescence peaks as the detection signal, reducing errors caused by light source fluctuations and probe concentration, significantly enhancing detection stability. These innovations address traditional fluorescence detection’s low sensitivity and poor anti-interference capabilities, offering solutions through material design and signal processing innovations [111,112].

#### 2.3.2. Applications of Fluorescence Spectroscopy in Pesticide Residue Detection

Fluorescence spectroscopy coupled with advanced data analysis techniques has demonstrated significant advantages in the detection of pesticide residues. Wang et al. developed a method for detecting carbamate pesticide residues (clothianidin and carbaryl) in tomatoes based on EEM fluorescence combined with a back-propagation neural network optimized by a mind evolutionary algorithm (MEA-BP). By dynamically matching the characteristic vibrational peaks of pesticides within the 8–12 μm wavelength range (e.g., P=O bond at 1240 cm^−1^) using an electrostatic gating technique and enhancing the signal with complex frequency waves (CFW), the method achieved a detection range of 0.01–1.00 μg/mL, with LOD as low as 0.147 μg/mL for clothianidin and 0.159 μg/mL for carbaryl. The average recovery rates ranged from 98.94% to 99.25%. The MEA-BP model outperformed the traditional BP network, reducing iteration time by 36% (304 iterations), decreasing mean square error by 66% (to 0.0057), and increasing the correlation coefficient to 0.99873. The study further validated that soaking in a sodium bicarbonate solution for 12 min was the most effective method for pesticide removal, reducing residue levels to 30.21–37.96%. This approach provides a highly sensitive, low-cost solution for food safety monitoring [113].

##### Application of NIR Fluorescent Probes in the Detection of Organophosphorus Pesticides

Alongside breakthroughs in conventional pesticide detection methods, the rapid development of NIR fluorescent probe technology has opened new avenues for detecting organophosphorus pesticides. Yi et al. developed a NIR fluorescent probe (Probe 1) based on a hemicyanine scaffold, in which hydroxyl groups were acylated with acetyl chloride and a fluorescence quenching group was introduced. The probe leverages the specific hydrolysis of ester bonds by carboxylesterase (CES), releasing a fluorophore that emits at 710 nm, thereby enabling the detection of OPPs [78]. The probe achieves a detection limit of 0.1734 μg/L for propoxur, with a linear range of 0–10 μg/L, and spiked recovery rates in cabbage samples ranging from 97.63% to 100.21% (RSD < 5.62%). Furthermore, Probe 1 has been successfully applied to real-time imaging in live cells (HeLa cells) and Staphylococcus aureus, demonstrating excellent sensitivity (detection limit of 0.0562 mU/mL for CES), high selectivity (stable performance within pH 6–7.5 and 36–40 °C), and outstanding biocompatibility (cell viability > 85%) [114].

Meanwhile, Zheng et al. designed a novel NIR fluorescent probe, PT-CES (NIR Fluorescent Probe for Carboxylesterase), based on a 4-[4-(dimethylamino)styryl] pyridinium scaffold and employing an ester hydrolysis mechanism. By harnessing an intramolecular charge transfer (ICT) effect, the probe enables ultrafast response to organophosphorus pesticides. It reaches signal stability within 30 min in PBS buffer, with a detection limit of 0.00396 mU/mL for CES. The detection range for dichlorvos (DCP) and trichlorfon extends up to 0–150 μg/L, with detection limits of 18.90 μg/L and 16.53 μg/L, respectively. The recovery rates in vegetable samples remain stable between 96.5% and 102.7%. PT-CES has not only been successfully used to detect pesticide residues in cucumbers but also validated through cellular co-localization experiments for its ability to target CES in mitochondria, enabling dynamic monitoring of CES activity in liver tissues. This provides an efficient and reliable analytical tool for both food safety and biomedical research [115].

In comparison, Probe 1 developed by Yi et al. enables the of propoxur at ultra-low concentrations, whereas PT-CES, designed by Zheng et al., offers faster response kinetics and a wider linear detection range. These technological advances not only offer new strategies for rapid, on-site detection of pesticide residues but also expand the potential applications in live-cell imaging and monitoring of enzyme activities related to diseases.

##### Application of Fluorescent Probes Based on Novel Functional Materials in Pesticide Detection

In recent years, the rapid development of novel functional materials such as nanomaterials, metal-organic frameworks (MOFs), quantum dots, and metallic nanoparticles has opened up innovative pathways for the design and performance enhancement of fluorescent probes [116,117,118]. Against this backdrop, Xu et al. developed a wearable glove sensor based on carboxymethyl cellulose (CMC) aerogels integrated with dual fluorescence centers {Europium-based MOFs (EuMOFs) red fluorescence/carbon dots blue fluorescence}. This sensor employs a ratiometric fluorescence strategy to achieve non-invasive detection of OPPs. The sensor is fabricated using a post-synthetic cation exchange method to produce EuMOFs with high fluorescence quantum yields (38%), which are then co-loaded with carbon dots that provide stable reference signals into a porous CMC aerogel matrix. The detection principle is based on the competitive adsorption between EuMOFs and the target compound chlorpyrifos (CP). Carbon dots serve as an internal reference signal to correct for environmental interference. The sensor responds within 30 s, achieving a detection limit for CP on agricultural product surfaces as low as 89 nM, with a linear range of 5–40 μM. Actual sample tests demonstrated excellent recovery rates (96.5–102.7%) and robust interference resistance. Notably, as pesticide concentrations increase, the sensor’s fluorescence color shifts visibly from red to blue, observable by the naked eye. Combined with a portable UV lamp, this allows for on-site interpretation. Featuring flexible, bendable design, high accuracy (R^2^ = 0.99529), and real-time detection capability, this device offers an innovative solution for food safety monitoring, integrating portability, sensitivity, and visual readability [119].

This advancement not only highlights the potential of integrating advanced functional materials into practical sensing devices but also underscores the importance of developing user-friendly tools for rapid, on-site analysis. The combination of high sensitivity, ease of use, and visual feedback makes this sensor particularly valuable for field applications in ensuring food safety and quality.

In addition to carbon dots, noble metal nanomaterials have also demonstrated unique advantages in pesticide detection. Wang et al. developed a colorimetric method based on the aggregation of unmodified AuNPs for the visual determination of total residues of nereistoxin-based insecticides. In this approach, various nereistoxin analogs in samples are first converted into nereistoxin—a bifunctional molecule containing both amino and thiol groups—through liquid-liquid extraction and alkaline hydrolysis. These functional groups can specifically bind to 13 nm citrate-stabilized AuNPs via electrostatic interactions and Au–S covalent bonding, inducing nanoparticle aggregation and a distinct color change from wine red to blue, clearly visible to the naked eye within the concentration range of 50–250 μg/kg. Quantitative analysis is achieved by measuring the absorbance ratio at 660 nm and 519 nm, with a detection limit of 40 μg/kg and a linear range of 50–250 μg/kg (R^2^ = 0.9953). The entire assay is completed within 3 min. Spiked recovery tests in real samples such as tea and kiwifruit yielded results between 61.1% and 105%, with coefficients of CV ≤ 10.9%, indicating reliable performance. When used in conjunction with a standard colorimetric chart, the method enables semi-quantitative visual detection, meeting Japan’s maximum residue limit (MRL) of 100 μg/kg for these pesticides. This strategy requires no expensive instrumentation, is simple to operate, and combines rapidity, low cost, and high specificity, showing strong potential for on-site applications [120].

##### Application of Fluorescence Sensors Based on Functional Materials in Pesticide Detection

Fluorescence spectroscopy technology has shown significant development potential and diversity in pesticide detection research, with key highlights being high sensitivity, rapid response, and good practical applicability [2,12]. Particularly through the integration of nanomaterials—such as AuNPs and up-conversion fluorescent materials—with biological recognition elements like aptamers, it has achieved specific capture and signal amplification for trace pesticides [121].

Based on the different sensing materials, these sensors can be mainly categorized into four types: enzyme-based fluorescent sensors, metal-organic framework (MOFs) sensors, graphene oxide (GO)-based sensors, and organic molecule sensors [122,123]. Enzyme-based fluorescent sensors combine biological enzymes such as carboxylesterase (PvCarE1) and alkaline phosphatase with nanomaterials like gold nanoclusters. By utilizing enzyme inhibition principles, they achieve detection of organophosphorus pesticides, offering high recovery rates and low detection limits. These sensors also provide semi-quantitative visualization capabilities. MOFs sensors leverage the tunable pore structures of MOFs and mechanisms such as fluorescence resonance energy transfer (FRET) for pesticide detection. However, their preparation processes are relatively complex, requiring precise control over synthesis conditions. GO-based sensors exploit the strong fluorescence quenching performance of GO or reduced GO (r-GO). They achieve detection through the adsorption and subsequent release of aptamers. Additionally, these sensors can be integrated with smartphone platforms to facilitate portable and on-site analysis. Organic molecule sensors are designed by coupling fluorophores, such as Boron-Dipyrromethene (fluorophore) (BODIPY), with recognition groups. They utilize photophysical mechanisms like photoinduced electron transfer (PET) to achieve highly selective detection. Some of these sensors have been further developed into test strips for field-deployable visual detection. Current challenges focus on material stability, large-scale production, and optimization of biocompatibility. Future directions will concentrate on integrating multiple technologies and expanding practical application scenarios. These advancements not only enhance the sensitivity and specificity of pesticide detection but also pave the way for developing more robust, user-friendly tools that can be widely applied in food safety monitoring and environmental protection.

Enzyme-based fluorescent sensors represent a typical paradigm of combining biological recognition with nanomaterials. Wang et al. developed a fluorescence sensor based on recombinant CES PvCarE1 and glutathione-stabilized gold nanoclusters (GSH-AuNCs) for the rapid detection of OPPs. The GSH-AuNCs were synthesized via a high-temperature reduction method, with an average particle size of 3.1 nm and exhibiting fluorescence at 630 nm when excited at 400 nm. The detection principle is based on OPs inhibiting the catalytic activity of PvCarE1 on p-nitrophenyl acetate (p-NPA), which hydrolyzes to produce p-nitrophenol (p-NP), quenching the fluorescence of GSH-AuNCs through an inner filter effect. Under optimized conditions (pH 7.5, 25 °C), the sensor achieved detection limits of 0.2 μg/L for DCP, 5 μg/L for trichlorfon, and 5 μg/L for profenofos, with linear ranges spanning from 0.2 to 200 μg/L to 5 to 1000 μg/L. In spiked apple samples, recoveries ranged from 84.5% to 106.3%, with RSD of 1.3% to 10.5%, demonstrating good accuracy and practicality [124].

Wei et al. reported a fluorescence sensor based on ALP-triggered in situ reactions for highly sensitive detection of OPPs. The material preparation involves ALP catalyzing the dephosphorylation of substrate Ascorbic Acid Phosphate (AAP) to generate ascorbic acid, which subsequently reacts with o-phenylenediamine (OPD) to form a fluorescent product, DFQ. The analytical method leverages the principle that OPPs inhibit ALP activity, leading to a decrease in fluorescence signals, detected via fluorescence spectroscopy (excitation/emission wavelengths: 360/425 nm). Detection performance showed a linear range of 20 pg/mL to 1000 ng/mL for chlorpyrifos, with a detection limit of 15.03 pg/mL (Signal-to-Noise Ratio = 3), outperforming most reported methods. In practical applications, the spiked recovery rates for leek and celery samples were between 94.5% and 106.7%, with RSD below 11.51%, validating the accuracy and reliability of the method. This sensor does not require synthesis of nanomaterials or complex labeling and can achieve semi-quantitative visual detection through changes in solution color under UV light [125].

Qvortrup et al. reviewed fluorescence-based detection methods for OPPs, with a focus on ALP-triggered reaction-based fluorescence sensors. Material preparation involves ALP catalyzing the conversion of substrate AAP into AA, which then reacts with OPD to form the highly fluorescent product DFQ, exhibiting an emission peak at 425 nm. OPPs reduce fluorescence signals by inhibiting ALP activity. Analytical methods indicated that the sensor has a linear range of 20 pg/mL to 1000 ng/mL for chlorpyrifos, with a detection limit as low as 15.03 pg/mL (S/N = 3), surpassing most reported methods. In actual samples (leek, celery), the recovery rates reached 94.5% to 106.7%, with RSD below 11.51%, and it also features semi-quantitative UV colorimetric functionality. This method does not require complex labeling or expensive instruments, achieving high sensitivity and visual detection through changes in fluorescence intensity and color [126].

Fu et al. reviewed sensors for detecting OPPs based on ALP-triggered in situ fluorescence reactions. Through ALP catalyzing the conversion of AAP to AA, which then reacts with OPD to form the strong fluorescent product DFQ (425 nm), OPPs reduce fluorescence intensity by inhibiting ALP activity. Under optimized conditions, the sensor had a linear detection range of 20 pg/mL to 1000 ng/mL for chlorpyrifos, with a detection limit as low as 15.03 pg/mL and recovery rates of 94.5% to 106.7% (RSD < 11.51%). This method does not require complex labeling or costly equipment and enables semi-quantitative detection through color changes under UV light, successfully applied to detect chlorpyrifos in leeks and celery, showcasing high sensitivity, broad detection range, and good applicability to real samples [127]. These enzyme-based sensors demonstrated good recovery rates of 84.5% to 106.7% in practical agricultural products, with RSD typically below 11.51%, confirming their analytical reliability.

MOF sensors have garnered significant attention due to their tunable pore structures and surface properties. Tu et al. reviewed the research progress of non-biological fluorescence sensors in pesticide detection, with a focus on summarizing sensor designs based on organic small molecules, MOFs, and nanomaterials. The material preparation methods include the synthesis of organic ligands, construction of MOFs (such as Zr-MOF and ZnPO-MOF), and quantum dot functionalization (e.g., CdTe QDs combined with thiourea). Analytical methods primarily rely on fluorescence enhancement/quenching mechanisms, such as PET and FRET. In terms of detection performance, these sensors exhibit high sensitivity towards various pesticides; for instance, Zr-MOF achieved a detection limit as low as 0.456 nM for methyl parathion, while CdTe QD sensors reached a detection limit of 0.1 nM for chlorpyrifos. The recovery rates in real samples (such as water and vegetables) were generally above 90%, with ZnPO-MOF showing recovery rates consistent with chromatographic results when detecting methyl parathion in irrigation water [128].

For example, Wang et al. innovatively constructed a biomimetic fluorescence sensing system based on palladium metal-MOFs (Pd-MOF). Nanoprobes with an average size of 260 nm were prepared through thermal polymerization, featuring a microporous structure of 2.0 nm and an ultra-narrow bandgap of 0.025 eV, which endows the system with molecular recognition capability for phorate. Upon target binding within the pores, P-π conjugation increases the bandgap to 0.046 eV, triggering fluorescence enhancement at 612 nm, achieving an ultra-low detection limit of 0.0017 ppb (a 20-fold improvement over national standards). This probe completes detection within 45 s, with recovery rates ranging from 87.69% to 106.12% in tap water and agricultural product samples. XANES spectroscopy and (DFT) confirmed the coordination mechanism between Pd and phorate. A biomimetic-designed test strip further enables rapid visual detection, offering a new solution for pesticide residue monitoring [129]. These sensors combine rapid response (within two minutes) and high selectivity but face challenges such as complex preparation processes or matrix interference for some materials. Future work should focus on optimizing stability and practicality. These advancements not only enhance the sensitivity and specificity of pesticide detection but also pave the way for developing more robust, user-friendly tools that can be widely applied in food safety monitoring and environmental protection.

GO and reduced GO (r-GO) based sensors have become a research hotspot due to their excellent fluorescence quenching performance. Sun et al. reviewed the application of fluorescence sensors based on GO and r-GO in the safety detection of aquatic products. The material preparation primarily involves methods such as the Hummers method for GO and chemical reduction, plasma reduction for r-GO. r-GO exhibits stronger fluorescence quenching capabilities due to its higher sp^2^ hybridized regions. Analytical methods are based on the principle of FRET, where fluorescently labeled aptamers interact with GO/r-GO through adsorption–desorption to detect target molecules. For pesticide detection, sensors achieved detection limits as low as 0.14 ppb (pure chlorpyrifos) and 2.05 ppb (commercial formulation). Detection of diazinon using upconversion nanoparticles modified aptamers reached a limit of 0.023 ng/mL, suitable for environmental and agricultural samples. Some sensors integrate with smartphone platforms or test strips for portable detection; for instance, Cd^2+^ detection using a fluorescent test strip combined with a smartphone has a detection limit of 0.1 mM. The PQ fluorescence quenching sensors exhibited satisfactory recovery rates in real water samples and Fe-GQDs-based sensors can be reused through magnetic separation. However, specific recovery rate data were not explicitly reported in the literature. Overall, GO/r-GO sensors exhibit high sensitivity, selectivity, and practicality, although large-scale production and biocompatibility still require optimization [130].

Yu et al. further reviewed the use of GO and r-GO-based fluorescence sensors in the safety detection of aquatic products. Material preparation methods include Brodie, Staudenmaier, and Hummers methods for synthesizing GO, along with chemical reduction, thermal reduction, and plasma reduction techniques for producing r-GO. Analytical methods leverage the fluorescence quenching properties of GO/r-GO based on FRET principles. Fluorescently labeled aptamers bind to target molecules, leading to the recovery of fluorescence signals for detection. In pesticide detection, sensors demonstrated detection limits as low as 0.13 nM to 4 ng/mL for OPPs like DCP and diazinon, with good recovery rates. Diazinon detection was applicable to both environmental and agricultural samples [131]. However, large-scale production and biocompatibility remain significant bottlenecks limiting their widespread application.

These advancements highlight the potential of GO and r-GO-based sensors for achieving high sensitivity and specificity in pesticide detection, but challenges such as scalability and biocompatibility need to be addressed to fully realize their potential in practical applications. These sensors offer promising solutions for enhancing food safety monitoring and environmental protection, provided that future research focuses on optimizing these aspects.

Organic molecule sensors have achieved highly selective detection through sophisticated molecular design. Sharma et al. reviewed various organic fluorescent sensors for pesticide detection. In terms of material preparation, the primary strategy involves coupling fluorophores—such as rhodamine, BODIPY, and naphthalimide—with specific recognition groups (e.g., hydroxamic acid, oxime) to enable detection via photophysical mechanisms like PET and ICT. The analytical approach relies on phosphorylation reactions that trigger changes in fluorescence signals; for example, a β-hydroxyoxime sensor undergoes cyclization to form an isoxazole, resulting in fluorescence enhancement, while BODIPY derivatives achieve signal amplification by restricting C-Ar bond rotation. These sensors exhibit excellent performance: a naphthalimide-based probe achieves a detection limit of 0.52 μM for fluoride ions, and a BODIPY sensor detects DCP down to 20.7 ppb. In agricultural products, certain sensors demonstrate recovery rates of 94.5–106.7% with RSD < 11.51%. Integration with test strip technology further enables visual, on-site detection [132]. Despite challenges related to complex synthesis, the tunable nature of their molecular structures offers broad opportunities for developing novel pesticide sensors.

##### Advances in Multimodal Pesticide Residue Detection Based on Fluorescence Spectroscopy

Recent studies have demonstrated that integrating fluorescence spectroscopy with other techniques can significantly enhance detection performance. Fen et al. developed a CES fluorescent probe (Probe 1) based on a benzothiazole fluorophore and a phenyl dimethylcarbamate recognition group for the rapid detection of carbamate pesticides. The probe was synthesized via a two-step organic route with a 79% yield. It operates through the enzymatic hydrolysis of the ester bond in the probe by CES, releasing the fluorescent benzothiazole moiety (emission at 436 nm). The system responds within 25 min in pH 7.0 buffer, achieving a detection limit of 0.02703 U/mL for CES. By employing a competitive enzymatic inhibition mechanism, the probe exhibits high sensitivity toward the representative carbamate pesticide carbaryl, with a detection limit of 27.40 nM and a linear range of 0–1.0 μM, along with excellent selectivity against 16 potential interfering substances. In real-sample analyses of fruit juices and vegetable juices, the recovery rates ranged from 97.85% to 103.10% (RSD < 1.44%). Notably, the method was innovatively coupled with smartphone-based RGB analysis, enabling visual quantification of a solution color gradient (from light to deep blue) by the naked eye. This approach provides a field-deployable, equipment-free solution for rapid on-site detection [133].

It is worth noting that although fluorescent probes on enzymatic hydrolysis enable efficient detection of individual pesticides, they are still limited in the simultaneously analyze multiple pesticide residues. Bian et al. developed a multi-pesticide residue detection method by combining fluorescence spectroscopy with a PLS regression model, overcoming the traditional limitation of conventional fluorescence techniques—which typically allow for detection of only one analyte due to overlapping emission spectra. The study selected four pesticides with highly overlapping fluorescence signals: zhongshengmycin, paclobutrazol (PBZ), boscalid, and pyridaben. By preparing 151 mixed samples with varying concentrations (e.g., zhongshengmycin: 0–0.0305 mg/mL, pyridaben: 0–0.0033 mg/mL), multiple PLS models were established. The approach (1) utilized a seven-principal-component model; after cross-validation and removal of outliers, the model achieved R^2^ values ranging from 0.9827 to 0.9913, with recovery rates approaching the theoretical value of 1; (2) eliminated the need for complex sample pretreatment, enabling simultaneous, non-destructive detection of multiple pesticides in water samples; and (3) achieved RMSE as low as 0.000107–0.000996 mg/mL, indicating sufficient sensitivity for real-world applications. This strategy provides a rapid, cost-effective approach for online water quality monitoring [134].

**Figure 4:**
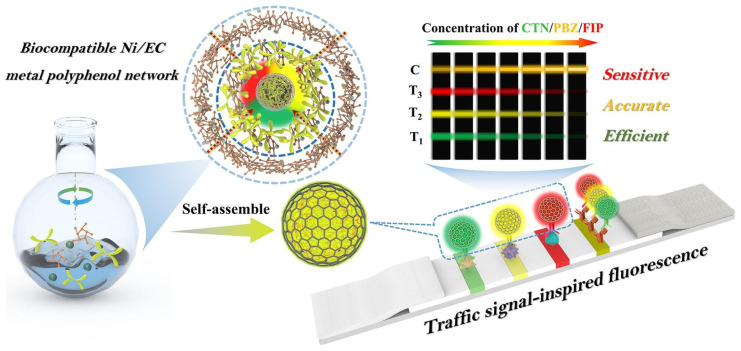
Traffic signal-inspired fluorescence lateral flow immunoassay utilizing self-assembled AIENP@Ni/EC for simultaneous multi-pesticide residue detection [135].

To expand the application of pesticide residue detection and meet the growing demand for multi-analyte screening in agricultural products, Zhang et al. developed an innovative high-throughput method by integrating fluorescent labeling with immunoassay technology, enhancing its compatibility with complex matrices (Figure 4). Specifically, they reported a traffic-light-inspired fluorescent lateral flow immunoassay (T-FLFIA) for the simultaneous detection of multiple pesticide residues. Through a self-assembly approach, three distinct fluorescent aggregation-induced emission nanoparticles (AIENP@Ni/EC) emitting green, yellow, and red light were synthesized. These nanoparticles, with an average size of 155–188 nm, feature a core structure composed of AIEgens encapsulated within a nickel-epicatechin (Ni/EC) metal–polyphenol network. Antibodies were then conjugated to the nanoparticles via electrostatic adsorption to form immunoprobes. Leveraging a competitive immunoassay format, the method enables simultaneous detection of chlorothalonil (CTN), paclobutrazol (PBZ), and fipronil (FIP) within 5 s, with linear ranges of 0.05–100, 0.05–250, and 0.05–10 ng/mL, respectively, and detection limits as low as 0.038, 0.025, and 0.046 ng/mL. In real spiked samples (apple and cowpea), the recovery rates ranged from 90.2% to 114.0%, with a qualitative accuracy of 92.5%. Compared to traditional gold nanoparticle-based methods, the sensitivity was improved by 3.37- to 5.52-fold. The method also demonstrated high specificity—no cross-reactivity with 12 potential interfering substances—and strong resistance to matrix effects [135].

Fluorescence spectroscopy has emerged as a powerful tool for pesticide residue detection, leveraging high sensitivity, rapid response, and diverse signal transduction mechanisms. Advances in fluorescent probes—ranging from NIR-emitting molecular designs to functional nanomaterials like MOFs, quantum dots, and GO—have enabled detection at trace levels (down to pg/mL), often with excellent selectivity and biocompatibility. Enzyme-based systems, particularly those exploiting ALP or CES inhibition, achieve remarkable sensitivity through signal amplification via in situ reactions, while ratiometric and smartphone-integrated platforms enhance portability and on-site applicability. Notably, multimodal strategies such as T-FLFIA and PLS-regression-enhanced spectroscopy overcome traditional limitations in multiplex detection, allowing for simultaneous quantification of multiple pesticides in complex matrices. Despite these advances, the technique remains constrained by the non-fluorescent nature of many pesticides, necessitating derivatization or indirect detection schemes, and is susceptible to environmental interference affecting signal stability. Moreover, while nanomaterial-based sensors offer superior performance, challenges in scalability, reproducibility, and biocompatibility persist. Thus, although current innovations significantly broaden the scope and practicality of fluorescence-based sensing, further research is essential to improve robustness and standardization for widespread real-world implementation.

Compared to infrared spectroscopy, research on fluorescence spectroscopy is still relatively limited and faces several challenges. Fluorescence spectroscopy can only detect substances that are intrinsically fluorescent, which significantly restricts its application scope. For non-fluorescent analytes, the method requires the addition of fluorescent reagents or derivatization reactions to generate a detectable signal, adding complexity and difficulty to pesticide residue analysis. Furthermore, fluorescence signals are highly susceptible to environmental factors such as solvent composition, pH, and temperature, which can affect fluorescence intensity and stability, potentially leading to inaccurate measurements. These limitations hinder the robustness and reproducibility of the technique. Consequently, substantial research and development efforts are still needed before fluorescence spectroscopy can be widely adopted in practical, real-world applications.

#### 2.3.3. Comparison of Various SERS Detection Methods Based on Fluorescence Spectrum

Table 2 highlights the diverse performance characteristics of various fluorescence spectroscopy-based methods in pesticide residue analysis. Fluorescence spectroscopy combined with biosensing technology demonstrates high recovery rates of 98.7–109.2% and an extremely low detection limit of <1.2 × 10^−12^ M for pesticides such as paraoxon and chlorpyrifos, with a detection time under 20 min, making it suitable for rapid detection of multiple pesticide classes [105]. For FIP, a fluorescent immunosensor achieved recoveries of 95.95–137.07%, a detection limit of 0.01 μg/L, and exceptionally high precision (RSD 0.07–0.23%) [110]. A biomimetic fluorescent sensor exhibited recoveries of 87.69–106.12% for demeton-S-methyl, a detection limit of 0.0017 μg/L, and a response time of only 45 s, combining high sensitivity with rapid analysis [99]. EEM fluorescence spectroscopy yielded recovery rates close to 99% for insecticides TSU and CBL, with detection limits of 0.147 and 0.159 μg/mL, respectively [114].

NIR fluorescent probes showed recoveries of 97.63–100.21% for isocarbophos, with RSDs of 2.32–5.62% and a detection limit of 0.030 μg/L [110]. Another NIR method reported recoveries of 96.50–101.83% for DCP and 97.09–102.71% for trichlorfon, although with higher detection limits (approximately 16–19 μg/L) [116]. Dual-signal fluorescence assays achieved an RSD ≤ 12% for chlorpyrifos, with a detection limit of 89 × 10^−9^ M and a response time of just 30 s [120]. Turn-on fluorescence methods enabled detection of DCP, trichlorfon, and profenofos within minutes, with recoveries ranging from 84.5% to 106.3% and detection limits of 0.2, 5, and 5 μg/L, respectively [124].

Although traditional fluorescence spectroscopy can take up to 180 min, it achieves a remarkably low detection limit of 0.015 ng/mL for chlorpyrifos, with stable recoveries of 94.5–106.7% [125]. Conversely, for demeton-S-methyl, this method reached an ultra-low detection limit of 1.7 pg/L within 45 s, underscoring its potential for high sensitivity [129]. Dual-excitation colorimetric techniques allow for simultaneous detection of four pesticides—including ATZ and carbaryl—within 5 min, with all recoveries exceeding 95% and RSDs below 3.5% [131]. Smartphone-based image recognition offers field-deployable convenience, achieving a detection limit of 27.40 × 10^−9^ M for carbaryl and recoveries of 97.85–103.10% [120]. The integration of PLS models with fluorescence spectroscopy is well-suited for online monitoring, supporting a broad concentration range (0–0.0305 mg/mL) [134]. Multicolor fluorescent signaling methods achieve detection limits as low as 0.025–0.046 ng/mL for CTN, PBZ, and FIP, with response times under 5 s, demonstrating exceptional speed and ultrahigh sensitivity [135]. Overall, these methods continue to improve in sensitivity, speed, and portability, providing diverse solutions for pesticide residue detection.

### 2.4. UV-Vis Spectroscopy

#### 2.4.1. Principle

UV-Vis spectroscopy is an analytical technique based on the selective absorption of electromagnetic radiation in the 200–800 nm wavelength range by molecules. The fundamental principle involves electronic transitions of valence electrons (σ, π, or n electrons) within a molecule, which absorb photons of specific energy and undergo transitions between electronic energy levels, such as σ→σ*, n→σ*, π→π*, or n→π*. These transitions generate characteristic absorption spectra [136,137]. The UV-Vis region is typically divided into near-UV (200–400 nm), visible (400–800 nm), and far-UV (10–200 nm, requiring vacuum conditions for detection). Transitions such as π→π* and n→π* commonly produce absorption bands in the near-UV to visible region, making them particularly useful for the analysis of organic compounds [52,138].

In pesticide residue detection, UV-Vis spectroscopic methods primarily include three approaches: UV spectrophotometry, derivatization-based colorimetric methods, and enzyme inhibition assays, collectively forming a multidimensional and synergistic detection framework. Spectrophotometry enables rapid screening through the characteristic absorption of target molecules, derivatization enhances sensitivity via chemical reactions, and enzyme inhibition improves specificity through biochemical recognition. Current research focuses on enhancing performance through nanomaterials, integration with microfluidic systems, and the development of intelligent detection devices, leading to significantly lower detection limits (reaching μg/kg levels), shorter analysis times (≤15 min), and improved suitability for on-site applications. These three methodologies complement each other, collectively addressing challenges such as matrix interference and poor enzyme stability, thereby providing multi-tiered solutions for pesticide residue monitoring.

#### 2.4.2. Applications of UV-Vis Spectroscopy in Pesticide Detection UV Spectrophotometry

UV-Vis spectroscopy is a vital analytical tool in pesticide detection, relying on either the characteristic absorption of target compounds at specific wavelengths or the chromogenic effects of their reaction products. Owing to its widespread instrument availability, methodological versatility, and cost effectiveness, this technique fulfills a broad range of analytical requirements—from high-precision laboratory analysis to rapid on-site screening. With technological advancements, modern UV-Vis methods have achieved significant improvements in sensitivity and selectivity through the synergistic design of optical signals with chemical or biological reactions, offering standardized solutions for pesticide residue monitoring.

Specifically, UV spectrophotometry has proven effective for pesticide residue screening in tea, owing to its simple instrumentation, low cost, and rapid analysis (approximately 30 min per test). Chen et al. developed an ultrasound-assisted extraction combined with UV spectrophotometry for the highly sensitive detection of orthophenylphenol residues in tea, achieving a detection limit of 0.12 μg/g. The method demonstrated a linear range of 0.5–20 μg/mL and spiked recovery rates of 90.1–91.2%, meeting the requirements for quantitative analysis. Compared to traditional chromatographic methods, this approach eliminates the need for complex sample preparation and expensive equipment, reducing the cost per sample by 65%. Furthermore, the coefficient of variation across six types of tea samples was less than 5%, demonstrating both rapid screening capability (30 min per sample) and accurate quantification. This cost-effective and efficient method provides a practical solution for agricultural product safety supervision [139].

Yu et al. prepared 84 apple juice samples spiked with varying concentrations of imidacloprid, ACE, and thiamethoxam, and employed UV spectroscopy (200–350 nm) combined with a sparrow search algorithm-optimized backpropagation neural network (SSA-BPNN) for simultaneous detection of these neonicotinoid pesticides. UV-Vis spectra were collected using a spectrophotometer and preprocessed with Savitzky-Golay smoothing to reduce noise before model construction. The results showed that the SSA-BPNN model outperformed other models (ELM, BPNN, PSO-BPNN), achieving determination coefficients (R^2^) of 0.9985–0.9994 and RMSE of 0.0501–0.0609 mg/L in the prediction set. The method requires no complex sample pretreatment and achieves detection limits below 0.6 mg/L (lowest tested concentration), making it suitable for rapid screening of multiple pesticide residues in apple juice. This approach offers an efficient and low-cost analytical solution for food safety monitoring [140].

Notably, the characteristic absorption of the P=O bond in OPPs at 210–230 nm provides a theoretical basis for their detection via UV spectrophotometry, enabling analysis within 5 min. However, this method is susceptible to matrix interference. Dong et al. developed an innovative chromogenic reaction system that enables simultaneous and accurate determination of OPPs such as methamidophos, omethoate, and dimethoate. The method exhibited a linear detection range of 0.1–10 mg/kg (R^2^ > 0.99) and consistent spiked recovery rates of 88.2–92.5% (RSD < 3.0%). Sensitivity was improved by 40% compared to traditional test strip methods. The method substantially simplifies sample preparation, reducing analysis time to just 15 min per sample and cutting overall costs by 70%. Particularly suitable for large-scale screening in grassroots laboratories, this method combines rapid response, economic efficiency, and reliable results. It has been successfully applied to the testing of 120 batches of commercially available vegetables, demonstrating its feasibility and practicality as a technical solution for agricultural product quality and safety supervision [141].

##### Derivatization-Based Colorimetric Technology

Simultaneously, derivatization-based colorimetric technology enables the precise detection of trace-level pesticides through chemical transformation, thereby complementing UV spectrophotometry by leveraging the unique advantages of each method. This technique converts pesticide molecules into derivatives with distinct coloration or characteristic UV absorption via specific chemical reactions. By exploiting functional group-specific reactions, such as the condensation of carbamates with ninhydrin to form a purple-blue product, the method enhances detection sensitivity up to 0.001 mg/kg.

In recent years, significant advancements have been achieved in the development of novel derivatizing reagents, their integration with microfluidic chips, and the deployment of portable detection systems.

(i) Development of Novel Derivatizing Reagents

The design and synthesis of highly reactive derivatizing reagents have substantially improved detection sensitivity. For instance, 9-fluorenylmethyl chloroformate, as a newly developed derivatizing agent, exhibits three times higher reactivity than conventional reagents, enabling more efficient coupling with target pesticide molecules and achieving an exceptionally low detection limit of 0.001 mg/kg. This innovation not only enhances sensitivity but also broadens the range of detectable pesticides [142]. Additionally, broad-spectrum reagents have been explored: 2,4-dinitrofluorobenzene is effective for OPPs [142], while 5-(4,6-dichlorotriazinyl) aminofluorescein (DTAF) can be used to detect carbamate pesticides such as carbaryl.

(ii) Microfluidic Chip Integration

Microfluidic technology miniaturizes traditional batch-wise derivatization processes onto chip-based channels. By precisely controlling fluid dynamic parameters—such as flow rate and mixing time—it enables accurate regulation of derivatization reaction conditions. Recent studies demonstrate that integrating microfluidics reduces the entire derivatization-detection process from the conventional 2 h to just 15 min while improving reaction efficiency by over 40%.

(iii) Portable Detection Systems

Jiang et al. developed a supramolecular fluorescent probe, AFL@ALB, which specifically binds to pyrethroid pesticides, producing a visible “yellow-to-green” color change within 10 s. The method achieves a detection limit as low as 1.0773 μM, surpassing EU regulatory standards by a factor of 13. By innovatively integrating smartphone-based RGB analysis, the cost per test is reduced to merely RMB 0.5 (approximately USD 0.07), with field measurement errors below 7.2% and a total assay time of only 3 min. More notably, they pioneered a PBA/CNF aerogel sensor capable of real-time, visual monitoring of gaseous pyrethroids (e.g., those released from mosquito coils), achieving a detection limit of 1.7378 μM. This system establishes a technological closed-loop of “colorimetric reaction–intelligent recognition–multi-scenario adaptability,” offering a portable solution with laboratory-grade accuracy for monitoring agricultural product safety and environmental health [143].

##### Enzyme Inhibition Assay

UV-Vis spectroscopy plays a central role in enzyme inhibition-based pesticide detection, primarily relying on the specific inhibition of acetylcholinesterase (AChE) by organophosphorus and carbamate pesticides. The detection process involves three key steps: First, under normal reaction conditions, AChE catalyzes the hydrolysis of a substrate (e.g., acetylcholine), generating chromogenic products such as thiocholine. Subsequently, these products react specifically with the chromogenic reagent 5,5′-dithiobis-(2-nitrobenzoic acid), forming a characteristic absorption peak at 412 nm. When target pesticides are present in the sample, they bind to the active site of AChE, thereby inhibiting its catalytic activity. By using a UV-Vis spectrophotometer to monitor real-time changes in the absorbance of the reaction system, the inhibition rate of enzyme activity can be accurately calculated, enabling quantitative analysis of pesticide residues. This approach skillfully combines the high specificity of biological recognition (>95%) with the convenience of spectroscopic detection, achieving high sensitivity down to 1 ng/mL (e.g., for glyphosate). This method is especially well-suited for rapid on-site screening of complex matrices like fruits and vegetables, offering an efficient and dependable technical solution for food safety monitoring [144,145].

The widespread application of nanozyme technology—especially graphene-based materials—offers dual advantages: an 80% reduction in detection cost and exceptional resilience under extreme environmental conditions. For example, Wei et al. developed a nanozyme sensor array based on heteroatom-doped graphene (nitrogen-doped NG, nitrogen-sulfur co-doped Nitrogen-Sulfur Co-Doped Graphene (NSG), and GO for the detection of aromatic pesticides. NG and NSG were synthesized via a high-temperature pyrolysis method and utilized for their peroxidase-like activity to catalyze the 3,3′,5,5′-Tetramethylbenzidine-H_2_O_2_ chromogenic reaction, with absorbance measured at 412 nm. When pesticides adsorb onto the graphene surface, they specifically inhibit the enzyme-mimicking activity, and the resulting absorbance changes are monitored by UV-Vis spectroscopy. This sensor array successfully distinguished five pesticides, lactofen, methyl fluroxypyr, bensulfuron-methyl, fomesafen, and diafenthiuron, with a detection limit as low as 5 μM, and its practicality was validated in soil samples. Molecular simulations revealed that pesticide adsorption onto active sites of graphene occurs via π-π stacking and hydrogen bonding. By combining the high stability of nanozymes with the multiplex detection capability of sensor arrays, this method provides a simple and cost-effective solution for pesticide residue detection in complex matrices [146]. Future efforts should focus on multi-enzyme cooperative recognition, nanomaterial surface modification, and optimization using machine learning algorithms to overcome limitations in detectable pesticide categories and enhance anti-interference capabilities in complex samples, thereby promoting large-scale applications in smart agriculture and food safety supervision.

The integration of nanomaterials has significantly enhanced the sensitivity of UV–Vis spectroscopy by amplifying optical signals through LSPR or catalytic effects—AuNPs, for instance, can boost sensitivity up to tenfold compared to conventional methods. In enzyme inhibition assays, nanozymes such as heteroatom-doped graphene not only reduce costs by 80% but also improve stability and enable multiplex detection via peroxidase-mimicking activity, making them highly suitable for field applications. In contrast, derivatization-based methods achieve ultra-low detection limits (e.g., 0.001 mg/kg) through selective chemical transformation, yet often require precise reaction control and longer processing times. While both approaches benefit from portability advances—such as smartphone-coupled readouts—enzyme inhibition assays demonstrate superior reproducibility (RSD < 3.0%) and faster response (within minutes), owing to their robust biological recognition mechanism. Consequently, enzyme-based methods exhibit stronger advantages in on-site screening where rapid, reliable, and cost-effective analysis is critical, whereas derivatization excels in sensitivity for trace-level quantification under controlled conditions.

However, UV-Vis spectroscopy has several limitations: it is applicable only to pesticides containing aromatic rings or conjugated systems (approximately 35% of common pesticides), while non-UV-absorbing pesticides require derivatization. Additionally, measurements are susceptible to environmental factors such as solvent polarity and pH. For instance, hydrogen-bonding effects in fenitrothion can cause shifts in absorption peaks. Current development trends include enhancing absorption signals using nanomaterials (e.g., AuNPs can improve sensitivity tenfold), miniaturizing spectrometers for field-based rapid testing, and integrating with the Internet of Things to build cloud-based spectral databases. Although UV-Vis spectroscopy is slightly less specific than fluorescence spectroscopy, its advantages of low equipment cost and operational simplicity ensure its continued importance in pesticide residue screening at grassroots and field levels.

### 2.5. HSI Technology

#### 2.5.1. Principle

HSI captures spectral information—reflectance or transmittance—across hundreds of narrow, contiguous wavelength bands from a target object, integrating this with spatial imaging to form a “three-dimensional data cube.” This enables dual-dimensional analysis of both chemical composition and spatial distribution of materials [147]. Utilizing grating or prism-based dispersive systems, HSI finely segments wavelengths within the visible to NIR (Vis-NIR) spectral range (400–2500 nm), allowing for simultaneous recording of continuous spectral signatures (e.g., vibrational absorption peaks of pesticide molecules) at each pixel and acquisition of the target’s geometric and morphological information. Compared to conventional analytical methods, its core advantages include non-contact, non-destructive detection, high information density (simultaneous acquisition of chemical and spatial data in a single scan), and dynamic monitoring capability [148]. For instance, drone-mounted HSI systems can evaluate pesticide spray uniformity at the field scale, while whole-fruit or leaf surface residues can be spatially mapped, avoiding sampling damage and undetected blind spots [149].

#### 2.5.2. Applications of HSI in Pesticide Detection

In pesticide residue detection, HSI significantly enhances detection efficiency and precision by leveraging its unique fusion of spectral and spatial information. The technology can extract characteristic spectral bands—such as the 900–1000 nm absorption features of organophosphorus pesticides—to generate two-dimensional heatmaps of pesticide concentration distribution. This allows for the precise identification of residue accumulation within specific areas like apple stem cavities or leaf stomata, thereby offering a visual basis for washing and sorting procedures. Additionally, its early-warning capability can detect subtle spectral changes in crops under pesticide stress (e.g., red-edge shifts caused by reduced chlorophyll content), offering risk alerts for over-application before visible symptoms appear.

The core advantage of HSI lies in its ability to simultaneously capture the spatial distribution of chemical components and spectral “fingerprint” features in a single scan [150]. In contrast to traditional liquid chromatography, HSI eliminates the need for sample grinding, extraction, or chemical reagents, avoiding both sample destruction and reagent consumption. This characteristic renders it especially apt for the real-time sorting of fresh agricultural products, including fruits and vegetables. Employing multi-band collaborative analysis not only addresses the limitations inherent in single-spectral methods but also enhances overall performance. As a result, significant research progress has been achieved in pesticide residue detection on leafy greens and other crops, including lettuce, spinach, and mulberry leaves [151,152].

For example, in detecting OPPs (e.g., chlorpyrifos) on lettuce, researchers employed a HSI system in the 400–1000 nm range. By extracting changes in reflectance at 550 nm (chlorophyll absorption valley) and 720 nm (red-edge region) and combining these with a PLS discriminant analysis (PLS-DA) model, the study successfully differentiated residue samples across a concentration gradient of 0.5–10 mg/kg, achieving a classification accuracy of 89% [153]. For pyrethroid pesticides (e.g., cypermethrin) in mulberry leaves, scholars utilized short-wave infrared spectroscopy (900–1700 nm) to capture vibrational absorption features of C-Cl bonds in pesticide molecules. Applying spectral angle mapping Spectral Angle Mapping, they successfully localized residue accumulation near leaf veins, achieving a detection limit as low as 0.2 mg/kg—10 times more sensitive than conventional enzyme inhibition assays [26].

NIR transmittance spectroscopy, renowned for its speed and non-destructive nature, has shown destructive benefits in detecting pesticide residues on lettuce leaves. Ge et al. innovatively integrated chlorophyll fluorescence spectral data, thereby markedly enhancing the accuracy of predictive models. The research team adopted a combined strategy of wavelet transform and MD-MCCV algorithms to effectively extract optimal wavelength features from spectral data. The experimental results showed that the fused model achieved a prediction accuracy of R^2^ = 0.987 and a RMSE of only 0.005, fully validating the reliability and practical value of this technology in quantitative pesticide residue analysis [153].

The successful application of these techniques in lettuce detection has provided an important reference for pesticide residue monitoring in other crops. Researchers have since turned their attention to mulberry leaves, a key economic crop. In their investigation of pesticide residues in mulberry leaves, Zhou et al. achieved a major breakthrough by integrating near-infrared HSI with gas chromatography. Through microscopic observation of pesticide effects using SEM, and employing advanced methods such as SPA to extract characteristic wavelengths, they constructed multiple linear regression (MLR) models. Among these, the SPA-MLR model performed exceptionally well (R_p_ = 0.859, RMSEP = 38.789). This technology’s key contribution lies in enabling visualized detection of pesticide residue distribution, offering a novel technical approach for real-time monitoring in agricultural production [26].

Ma et al. proposed a non-destructive method for detecting pesticide residues on cantaloupe surfaces based on short-wave infrared HSI (SWIR-HSI, 1000–2500 nm) and an adaptive t-distribution honey badger algorithm-optimized extreme learning machine (tHBA-ELM). By comparing three models—ELM, Support Vector Machine (SVM), and PLS-DA—they found that ELM achieved the highest accuracy (79.5%) in identifying five pesticide types, including ACE and malathion. After normalization preprocessing, accuracy improved to 82%. The study innovatively applied the tHBA algorithm to optimize ELM parameters, enhancing population diversity through adaptive t-distribution mutation. This resulted in a test set accuracy of 93.5%, precision of 93.73%, and F1-score of 0.9355 for the tHBA-ELM model, outperforming GA-ELM (88.5%) and HBA-ELM (90.5%). The experimental results indicated that the SWIR band is sensitive to organic pesticides due to vibrational characteristics of C-H and O-H bonds, with the highest recognition rates for ACE and difenoconazole (95%), while these were slightly lower for cypermethrin (92.5%) due to its volatility. This method provides an efficient, non-destructive solution for pesticide detection on thick-skinned fruits and vegetables, though model parameters need to be tailored to different pesticides and cultivars to improve generalization [154].

With ongoing advancements in research, scientists are progressively investigating detection methodologies tailored to increasingly intricate situations. The demand for detecting mixed pesticide residues has propelled the evolution of HSI even further. Sun et al. demonstrated outstanding performance in detecting mixed pesticide residues on lettuce leaves by integrating hyperspectral sensing with imaging capabilities. This technique not only simultaneously acquires spectral and spatial information from samples but also enables precise selection of characteristic wavelengths through algorithms such as CARS and Recursive Feature Elimination based on Random Forest (RF-RFE). By establishing a Least Squares Support Vector Regression model and simplifying it with the Successive Projections Algorithm (SPA), detection efficiency was significantly enhanced. The high efficiency and accuracy of this non-destructive method represent a revolutionary technological upgrade for monitoring systems of agricultural product quality and safety [27].

Moreover, the incorporation of deep learning techniques with hyperspectral imaging data has facilitated the simultaneous identification and classification of various mixed pesticides, with accuracy rates surpassing 92%. Xu et al. employed both visible/NIR (Vis-NIR) (376–1044 nm) and NIR (915–1699 nm) HSI systems (HIS) to detect pesticide residue levels. Three different grape varieties were sprayed with four levels of pesticide application. Classification models for pesticide residue levels were developed using Logistic Regression (LR), SVM, Random Forest (RF), CNN, and Residual Neural Network (ResNet). Significance maps from CNN and ResNet were used to visualize the contribution of different wavelengths. Overall, the results obtained using NIR spectroscopy outperformed those from Vis-NIR. For Vis-NIR data, ResNet delivered the best performance, achieving an accuracy over 93%. For NIR data, LR performed best with accuracy exceeding 97%, although SVM, CNN, and ResNet also yielded close and robust results. The significance maps of CNN and ResNet revealed similar and consistent key wavelength ranges. These results indicate that deep learning models generally outperform traditional machine learning approaches. The study demonstrates that combining HSI with machine learning enables effective detection of pesticide residue levels in grapes [155].

**Figure 5:**
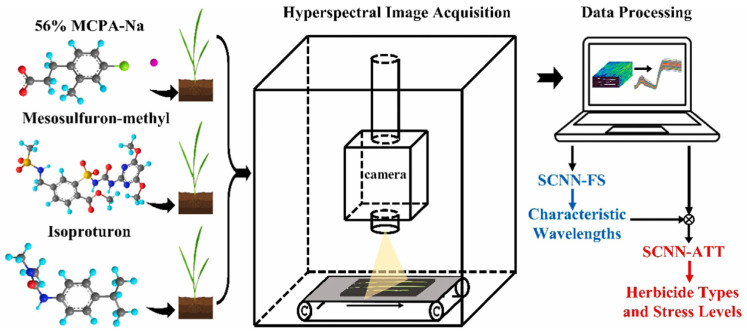
HSI of SCNN for predicting early herbicides in wheat varieties [156].

Liu et al. developed a rapid, non-destructive method for detecting early herbicide stress in wheat seedlings by combining visible/NIR HSI (Vis/NIR HSI) with a shallow CNN (SCNN) (Figure 5). Two wheat cultivars (HM-920 and XN-20) were grown in a greenhouse to the three-leaf stage and then treated with three herbicides—56% MCPA-Na, mesosulfuron-methyl, and isoproturon—at varying concentration gradients (33%, 67%, and 100% of the recommended dose). Hyperspectral data (400–1000 nm) were collected from the leaves using a HSI system. First-derivative analysis and the SCNN-ATT model were applied for feature extraction and classification. The results showed that spectral differences were primarily concentrated in the 518–531 nm (carotenoids), 637–675 nm (chlorophyll), and red-edge regions (around 700 nm). The SCNN-ATT model achieved a 96% accuracy in classifying herbicide types and approximately 80% accuracy in classifying stress levels within 48 h post-treatment. After selecting 15 key wavelengths using SCNN-FS (Feature Selection), the model maintained high accuracy, demonstrating the method’s capability for non-destructive identification of early herbicide stress and providing a technical foundation for real-time, in-field monitoring [156].

The ultimate goal of technological breakthroughs is to translate laboratory achievements into real-world applications. Researchers are increasingly focusing on how to move these advanced technologies out of the lab and into broader market use. In applied research within the 950–1650 nm spectral range, Ge et al. achieved a significant advancement by employing NIR transmittance spectroscopy. By combining Savitzky-Golay smoothing and SNV preprocessing with CARS and Iteratively Retains Informative Variables feature selection, and using a Grey Wolf Optimizer-optimized Support Vector Machine (SVM) model, the method achieved 100% accuracy in qualitative detection of fenvalerate and 98.33% accuracy for chlorpyrifos. This study not only validated the reliability of the technology but, more importantly, provided a practical and feasible technical pathway for developing portable pesticide residue detection devices, signaling a new era of opportunities in the field of food safety inspection [157].

## 3. Features, Research Limitations and Future Directions of Spectroscopic Techniques

### 3.1. New Features and Proposals

Spectroscopic detection technologies exhibit significant advantages in pesticide residue analysis, characterized by rapidity, non-destructiveness, and high sensitivity. When integrated with emerging materials and multimodal strategies, they demonstrate innovative features. Traditional spectroscopic methods, limited by single-feature analysis, have been surpassed by modern approaches that fuse multi-source data. For instance, near-infrared transmittance spectroscopy combined with chlorophyll fluorescence, optimized by wavelet transform and machine learning algorithms (e.g., WT-MD-MCCV), significantly enhances model accuracy (R^2^ = 0.987), enabling precise quantification of pesticide residues in lettuce. Additionally, HSI coupled with chromatography and SEM microstructure analysis, along with SPA-based feature wavelength extraction, establishes MLR models, offering a new paradigm for complex matrices like mulberry leaves. A novel perspective lies in intelligent detection mechanisms and multifunctional integration. Nanozyme-based sensor arrays leverage heteroatom-doped graphene’s peroxidase-like activity; pesticide adsorption inhibits catalytic reactions, achieving detection limits as low as 5 μM while distinguishing multiple aromatic pesticides. Molecular simulations reveal π-π stacking and hydrogen bonding as key interaction mechanisms. This approach reduces costs by 80% and exhibits strong environmental resilience, ideal for on-site screening. Meanwhile, perovskite-based SEIRA achieves an ultra-low detection limit of 23 fM—far below EU standards—by extending carrier lifetime via magnetic regulation and plasmonic enhancement. With recovery rates of 90.7–105.6% (RSD < 5.3%) and reusability, it combines high accuracy with practicality. These advances signify a shift toward ultra-sensitive, intelligent, and field-deployable spectroscopic technologies.

### 3.2. Intrinsic Limitations and Deep Bottlenecks

HSI enables non-destructive acquisition of high-dimensional spatial-spectral information, yet faces significant engineering challenges. Although HSI captures hundreds of continuous spectral bands from crop surfaces, its massive data volume demands high computational power and complex algorithms for dimensionality reduction and classification. More critically, its performance is highly sensitive to ambient lighting—variations in sunlight intensity, incident angle, shadows, and atmospheric scattering cause spectral signal drift, severely undermining model stability in field conditions. Moreover, HSI only probes surface-level information, making it ineffective for detecting systemic pesticides that have penetrated plant tissues. Additionally, models developed under specific instrument, crop, or environmental conditions often lack transferability, significantly limiting large-scale deployment.

SERS offers single-molecule-level sensitivity but suffers from a “reproducibility crisis.” Signal enhancement relies on “hot spots” generated by noble metal nanostructures (e.g., gold, silver), yet current fabrication methods—typically based on random nanoparticle assembly—result in substrates with inconsistent size, shape, and distribution. This leads to enhancement factors varying by orders of magnitude across batches, compromising quantitative accuracy and making standardized calibration nearly impossible [158,159]. Furthermore, matrix components such as proteins and sugars in agricultural samples readily adsorb onto substrates, causing signal interference or quenching. Silver substrates are prone to oxidation, while gold substrates are costly, and both face long-term stability issues, severely restricting reliable application in complex real-world scenarios.

Fluorescence spectroscopy is constrained by severe background interference and a narrow applicability range. Endogenous fluorophores in agricultural products—such as chlorophyll, flavonoids, and alkaloids—emit signals that often overlap with those of target pesticides, creating strong “autofluorescence” that masks low-concentration analytes [160]. Moreover, most pesticides are not intrinsically fluorescent (e.g., organophosphates, neonicotinoids), requiring derivatization or labeling to introduce fluorophores, which increases operational complexity and error risk. Fluorescence signals are also highly sensitive to environmental factors such as pH, solvent, and temperature, and are susceptible to photobleaching and quenching, leading to signal instability. These factors collectively hinder the achievement of high selectivity and reliability in complex matrices.

### 3.3. Systemic Challenges and Multidisciplinary Pathways to Breakthroughs

Current spectroscopic technologies face several cross-cutting systemic challenges. First, there is a lack of certified reference materials for diverse agricultural matrices, making instrument calibration and method validation difficult without traceable standards, thus undermining result credibility and inter-laboratory comparability. Second, sample pretreatment protocols—such as extraction and purification—lack standardization, with significant variations across laboratories. Matrix effects are pronounced, severely compromising reproducibility. More critically, existing techniques struggle to adapt to emerging pesticide types, such as bio-pesticides and nano-pesticides. These novel formulations have complex structures, weak or dynamic spectral features, and degradation pathways that current databases and algorithms cannot reliably identify, creating monitoring blind spots. Additionally, the absence of a unified, open-access global spectral database results in data silos, impeding the training and dissemination of AI-driven models.

Future breakthroughs must rely on systemic innovation through deep multidisciplinary integration. Materials science must develop stable, mass-producible SERS substrates—such as via electron-beam lithography or controlled self-assembly—to precisely engineer “hot spot” distribution. Photonics and micro-nanofabrication should advance miniaturization and portability of HSI and fluorescence devices, integrating MEMS spectrometers with edge AI chips for real-time, on-site analysis. Artificial intelligence can be leveraged to build illumination-adaptive models, spectral denoising algorithms, and cross-platform transfer learning frameworks. Simultaneously, environmental chemistry must investigate pesticide behavior and degradation pathways in various matrices to inform spectral recognition. Only by breaking down disciplinary silos and achieving synergistic evolution across “materials–devices–algorithms–chemistry–systems” can spectroscopic technologies transition from laboratory curiosities to practical field deployment.

### 3.4. Multimodal Integration and Intelligent, Sustainable Ecosystem

Establishing a hierarchical “screening–confirmation” multimodal detection system is key to achieving efficient and reliable monitoring. A three-tier architecture can be designed: Level 1 employs hyperspectral imaging for rapid, large-area screening to identify spectrally anomalous regions; Level 2 uses portable SERS or fluorescence devices for high-sensitivity chemical fingerprinting of suspicious samples; Level 3 involves laboratory-based mass spectrometry (e.g., GC-MS/LC-MS) for final quantification and regulatory confirmation. This “from broad to precise” strategy balances detection speed, cost, and accuracy, making it suitable for end-to-end monitoring from farm to market. Furthermore, integrating SERS or fluorescence modules with microfluidic chips enables automated sample processing and detection, enhancing workflow standardization and usability.

The future calls for an intelligent detection ecosystem driven by data, secured by blockchain, and accessible to all. A globally accessible, open spectral database should be established, integrating reference spectra across instruments, matrices, and concentrations, with standardized metadata to support model sharing and federated learning for continuous AI model optimization. Blockchain technology can immutably record the entire detection process—from sampling to reporting—ensuring full traceability and enhancing regulatory trust. At the same time, low-cost, solar-powered portable devices, coupled with smartphone interfaces, should be developed to empower smallholder farmers and consumers to participate in food safety monitoring. Ultimately, spectroscopic technologies will evolve beyond laboratory tools into intelligent sensing networks embedded within smart agriculture and food safety systems, providing robust technical support for global sustainability.

## 4. Conclusions

Spectroscopic techniques have evolved into a multi-layered technological framework for pesticide residue detection, with distinct application scenarios and performance advantages. This layered architecture—spanning from handheld field sensors to integrated lab-on-a-chip systems—reflects a paradigm shift in food safety monitoring: from reactive testing to proactive, real-time surveillance. SERS, leveraging plasmonic enhancement from nanomaterials and the development of portable devices, has become the preferred method for rapid on-site screening of trace pesticides—particularly suitable for the field detection of organophosphorus compounds. Recent advances in anisotropic gold nanostars and silver nanocubes have enabled enhancement factors exceeding 10^8^, allowing for detection at sub-ppb levels in complex agricultural matrices. Moreover, the integration of SERS with smartphone-based readout systems has empowered farmers and inspectors in remote areas to perform real-time analysis, transforming SERS into a democratized tool for grassroots food safety assurance. ATR-IR spectroscopy and fluorescence spectroscopy excel in laboratory settings: ATR-IR enhances selectivity toward carbamate pesticides via surface enhancement strategies, particularly when combined with molecularly imprinted polymers that act as synthetic receptors to pre-concentrate target analytes. Meanwhile, fluorescence spectroscopy enables simultaneous identification of multiple pesticide residues in complex matrices using NIR fluorescent probes. UV-Vis derivatization colorimetric methods, valued for their low cost, support routine testing in grassroots laboratories and community health centers. Recent innovations in enzyme-mimicking nanomaterials (nanozymes) have replaced traditional horseradish peroxidase in colorimetric assays, enhancing stability under ambient conditions. HSI, by fusing spatial and spectral information, can visually map the distribution patterns of pesticide residues on produce such as strawberries, apples, and leafy greens. This capability is transformative for quality control in post-harvest processing, where HSI systems integrated into conveyor lines can flag contaminated batches in real time. Nanomaterials, serving as key enhancing substrates, have significantly improved the sensitivity and selectivity of these detection methods. From plasmonic gold films to magnetic graphene oxide composites, engineered nano-materials not only amplify signals but also enable targeted enrichment, separation, and anti-interference functionalities. However, current technologies still face bottlenecks including insufficient stability of nanomaterials, limited coverage of standard spectral libraries, and the complexity of multi-technique integration. Future research should prioritize three key directions: first, developing intelligent responsive nanosubstrates to enhance the reproducibility of SERS in complex environments; second, constructing cross-modal spectral databases integrated with deep learning to enable rapid identification of unknown pesticides; and third, advancing the miniaturization of spectroscopy-MS (e.g., SERS-MS or HSI-MS) hybrid instruments to create seamless detection chains—from on-site screening to laboratory confirmation. These innovations will significantly improve the efficiency of agricultural product quality and safety monitoring, reduce non-point source pollution in agriculture through precise application guidance, and provide robust technological support for sustainable and green agriculture.

## Figures and Tables

**Table 1:** Detection performance comparison of various detection methods.

Detection Scheme	Data Analysis Methods	Detection Matrix	Pesticides	Recovery Rate (%)	RSD (%)	LOD	Detection Time	Reference
SERS	Portable Raman spectrometer (785 nm)	surface of fruit	Thiram	-	2.29	1 ppm	5–10 min	[54]
SERS	Portable Raman spectrometer	Milk	Melamine; Thiram	Melamine in milk: 95.9; Thiram in apple juice: 94.8	Melamine: 3.53; Thiram: 4.49	Melamine: 7.38 μg/L; Thiram: 86.1 μg/L	<10 min	[56]
SERS with stoichiometry	-	Citrus	TBZ	Good recycling performance	<10	0.1 mg/kg	<10 min	[57]
SERS	Portable Raman spectrometer	Tea	ACE; Thiram	ACE: 90.2–122.12 (apple juice), 89.86–117.23 (orange juice); Thiram: 90.38–113.42 (apple juice), 91.46–108.72 (orange juice)	ACE; Thiram	ACE: 0.272 mg/L (apple juice), 0.47 mg/L (orange juice); Thiram: 0.018 mg/L (apple juice), 0.025 mg/L (orange juice)	-	[58]
SERS with stoichiometry	SPA-PLS	Wheat	Deltamethrin	96.33–109.17	<5	0.16 μg/kg	-	[61]
SERS	Portable Raman spectrometer	Apple; Orange	ACE; Cypermethrin	ACE: 93.86–105.64; Cypermethrin: 92.62–102.3	-	ACE: 0.27 μg/kg; Cypermethrin: 1.71 μg/kg	-	[67]
SERS with stoichiometry	-	Apple	Thiram	88.32–111.80	2.92–4.91	0.020 mg/L	-	[68]
SERS	-	Lettuce; Cucumber	Methyl-parathion; Thiram; Chlorpyrifos	MP: 81.77–118.67; TMTD: 64.68–117.20; CPF: 73.10–126.80	8.58 (1078 cm^−1^), 9.29 (1583 cm^−1^)	MP: 0.072 ng/cm^2^; Thiram: 0.052 ng/cm^2^; Chlorpyrifos: 0.059 ng/cm^2^	-	[70]
SERS	Confocal Laser Scanning Raman Microscope System (InVia Reflex, Renishaw, UK)	Orange juice; Grapefruit juice; Milk	Carbaryl	82–99.8	-	-	-	[71]
SERS	Confocal Laser Scanning Raman Microscope System (Renishaw, UK)	Apple juice; Peach juice	TBZ	95–101	-	TBZ: 0.032 ppm (Apple juice), 0.034 ppm (Peach juice)	<30 min	[72]
SERS with stoichiometry		Tea	Pymetrozine; Thiram	-	<8%	0.0001 μg/mL	-	[74]
SERS with stoichiometry	GC-MS	Tea	Chlorpyrifos (CPS)	-	-	-	-	[76]
SERS with CNN Model		Tea	Thiram; Pymetrozine	Thiram: 83.06–92.6; Pymetrozine: 95.32–110.38	Thiram: 6.43; Pymetrozine: 10.41%	Thiram: 0.286 ppb; Pymetrozine: 29 ppb	-	[77]
SERS	Portable Raman spectrometer (785 nm)	Agricultural products	ACE; Thiacloprid	ACE: 85.1–107.6 (pear), 86.1–98.2 (Peach); Thiacloprid: 90.1–105.6 (pear), 73.5–112.8 (Peach)	7.5	ACE: 68.8 ng/g (pear), 33.7 ng/g (Peach); Thiacloprid: 36.4 ng/g (pear), 23.7 ng/g	-	[81]
SERS-MIPs	-	Rice	PCNB	94.4–103.3	4.6–7.4	0.005 µg/mL	-	[82]
SERS with MIPs	Portable Raman spectrometer (RT5000, 785 nm)	Wheat; Rice	Simazine; Prometryn	Simazine: 72.7–90.9; Prometryn: 79.1–86.5	Simazine: 1.7–7.6; Prometryn: 2.3–7.8	0.05 μg/mL	-	[83]
SERS	SPLD-RAMAN spectrometer (785 nm)	Food matrices	2,4-D	93.5–102.2	-	0.00147 ng/mL	2 h	[84]
SERS	Portable Raman spectrometer (785 nm)	-	Thiram; Carbendazim; TBZ; Carbaryl	--	4.65	<10^−15^ mol/L	-	[87]

**Table 2:** Detection performance comparison of various detection methods based on Fluorescence spectrum.

Detection Scheme	Data Analysis Method	Detection Matrix	Pesticides	Recovery Rate (%)	RSD (%)	LOD	Detection Time	Reference
Fluorescence spectrum with biosensor	Fluorescence imaging system	Apple	Paraoxon; DCP; Parathion; Deltamethrin	98.7–109.2	5.46–14.80	<1.2 × 10^−12^ M	<20 min	[105]
Fluorescence spectrum with biosensor	Fluorescence immunosensor	Spinach; Celery; Pak-choi	FIP	95.95–137.07	0.07–0.23	0.01 μg/L	-	[110]
Fluorescence spectrum	Bionic fluorescence sensor	Aquatic products	Phorate	87.69–106.12	<11.16	0.0017 μg/L	45 s	[111]
Fluorescence Spectroscopy with EEM	Fluorescence spectrophotometer (250–600 nm)	Cabbage	Tsumacide; Carbaryl	TSU: 98.94; CBL: 99.25	-	TSU: 0.147 μg/mL; CBL: 0.159 μg/mL	<dozens of minutes	[114]
NIR Fluorescence	NIR Fluorescent Prob (CES-targeted probe 1)	Cucumber	Isocarbophos; Other typical OPs	97.63–100.21	2.32–5.62	0.030 μg/L	Dozens of minutes	[115]
NIR Fluorescence	-	Milk; Strawberries; Grapes; Blueberries	DCP; Trichlorfon	DCP: 96.50–101.83; Trichlorfon: 97.09–102.71	-	DCP: 18.9 μg/L; Trichlorfon: 16.529 μg/L	-	[116]
Dual-signal fluorescence spectrum	Glove sensor (365 nm)	Tea; Kiwifruit; Rice; Cabbage;	Chlorpyrifos	-	≤12	89 × 10^−9^ mol/L	30 s	[120]
Fluorescence Spectroscopy	Fluorescence Spectrophotometer (425 nm)	Vegetables	Chlorpyrifos	94.5–106.7	<11.51	0.015 ng/mL	180 min	[125]
Fluorescence Spectroscopy	-	Pear; Cabbage; Pear juice; Cabbage juice	Phorate	87.69–106.12	<11.16	1.7 pg/L	45 s	[129]
Fluorescence Spectroscopy	Dual-color excited fluorescent sensing technology (310 nm and 360 nm)	Pesticide-containing aqueous solution	ATZ; Carbaryl; Chlorpyrifos; Prometryn	ATZ: 96.0–104.0; Carbaryl: 95.5–103.5; Chlorpyrifos: 997.0–105.0; Prometryn: 96.5–104.5	ATZ: 1.8–3.2; Carbaryl: 2.1–3.5; Chlorpyrifos: 1.6–3.0; Prometryn: 1.9– 3.3	ATZ: 0.091 μmol/L; Carbaryl: 0.103 μmol/L; Chlorpyrifos: 0.087 μmol/L; Prometryn: 0.095 μmol/L	<5 min	[131]
Fluorescence spectrum with Smartphone Image Recognition	-	Orange juice; Vegetable juice	Carbaryl	97.85–103.10	0.64–1.44	27.40 × 10^−9^ mol/L	Dozens of minutes	[133]
Fluorescence spectrum with PLS model	LS55 Fluorescence Spectrometer (Perkin Elmer)	Pesticide-containing aqueous solution	-	-	-	0–0.0305 mg/mL	Suitable for online monitoring	[134]
Multicolor fluorescence signal	-	Apple; Cowpea	CTN; PBZ; FIP	-	-	CTN: 0.038 ng/mL; PBZ: 0.025 ng/mL; FIP: 0.046 ng/mL	<5 s	[135]

## Data Availability

The original contributions presented in this study are included in the article. Further inquiries can be directed to the corresponding authors.

## Abbreviations

AAP	Ascorbic Acid Phosphate
ACE	acetamiprid
AgNPs	Silver Nanoparticles
ATZ	Atrazine
ATR-IR	Attenuated Total Reflection Infrared (Spectroscopy)
Au-Ag OHCs	Au-Ag cages
AuNPs	Gold Nanoparticles
AuNRs	Gold Nanorods
Au@Ag NRs	Gold Core-Silver Shell Nanorods
ACA	Aptamer for Acetamiprid
BPNN	Backpropagation Neural Network
BODIPY	Boron-Dipyrromethene (fluorophore)
CARS	Competitive Adaptive Reweighted Sampling
CdTe QDs	Cadmium Telluride Quantum Dots
CTN	Chlorothalonil
CV	Crystal Violet
Cu_2_O@Ag/Au@Ag	Cuprous Oxide-Silver Core-Shell and Gold-Silver Core-Shell Nanostructures
DCP	Dichlorvos
DFT	Density Functional Theory
DMD	Dithiocarbamate
2,4-D	2,4-Dichlorophenoxyacetic Acid
EC	Electrochemistry/Epicatechin
EEM	Excitation-Emission Matrix
EGDMA	Ethylene Glycol Dimethacrylate
ELM	Extreme Learning Machine
EuMOFs	Europium-based Metal-Organic Frameworks
FIP	Fipronil
FRET	Fluorescence Resonance Energy Transfer
GA	Genetic Algorithm
GA-ELM	Genetic Algorithm-Optimized Extreme Learning Machine
GO	Graphene Oxide
GSH-AuNCs	Glutathione-Stabilized Gold Nanoclusters
HBA-ELM	Honey Badger Algorithm-Optimized Extreme Learning Machine
HPLC	High-Performance Liquid Chromatography
HSI	Hyperspectral Imaging
ICT	Intramolecular Charge Transfer
LOD	Limit of Detection
LR	Logistic Regression
LSPR	Localized Surface Plasmon Resonance
MC	Mean Centering
MEA-BP	Mind Evolutionary Algorithm-optimized Back-Propagation
MIPs	Molecularly Imprinted Polymers
Mag@MIP NPs	Magnetic Molecularly Imprinted Polymer Nanoparticles
MOFs	Metal-Organic Frameworks
MS	Mass Spectrometry
MRLs	Maximum Residue Limits
NSG	Nitrogen-Sulfur Co-Doped Graphene
PAN	Polyacrylonitrile
PCA	Principal Component Analysis
PET	Photoinduced Electron Transfer
PLS	Partial Least Squares
PLSR	Partial Least Squares Regression
PSS	Polystyrene sulfonate
Pd-MOF	Palladium-based Metal-Organic Framework
PBA/CNF	Phenylboronic Acid/Cellulose Nanofiber
QuEChERS	Quick, Easy, Cheap, Effective, Rugged, and Safe
RF	Random Frog
RF-RFE	Recursive Feature Elimination based on Random Forest
RMSE	Root Mean Square Error
RMSEP	Root Mean Square Error of Prediction
r-GO	Reduced Graphene Oxide
Rp	Prediction Correlation Coefficient
RSD	Relative Standard Deviation
R^2^	Coefficient of Determination
SCNN	Shallow Convolutional Neural Network
SCNN-FS	Shallow Convolutional Neural Network for Feature Selection
SEIRA	Surface-Enhanced Infrared Absorption (spectroscopy)
SEIRAS	Surface-Enhanced Infrared Absorption Spectroscopy
SERS	Surface-Enhanced Raman Spectroscopy
siPLS	Synergy Interval Partial Least Squares
SNV	Standard Normal Variate
SPE	Solid-Phase Extraction
SWIR-HSI	Short-Wave Infrared Hyperspectral Imaging
tHBA-ELM	Adaptive t-Distribution Honey Badger Algorithm-Optimized Extreme Learning Machine
T-FLFIA	Traffic-Light-Inspired Fluorescent Lateral Flow Immunoassay
T-SEIRA	Transmission-mode SEIRA
T-SEIRAS	Transmission Surface-Enhanced Infrared Absorption Spectroscopy
TBZ	Thiabendazole
TMB	3,3′,5,5′-Tetramethylbenzidine
UV	Ultraviolet
UV-Vis	Ultraviolet-Visible
Vis-NIR	Visible-Near Infrared
ZOA	ZnO/Ag Heterostructures
μ-PADs	Microfluidic Paper-Based Analytical Devices
